# Distinct Features of Sedimentary Archaeal Communities in Hypoxia and Non-Hypoxia Regions off the Changjiang River Estuary

**DOI:** 10.1128/spectrum.01947-22

**Published:** 2022-09-06

**Authors:** Dayu Zou, Hongliang Li, Ping Du, Bin Wang, Hua Lin, Hongbin Liu, Jianfang Chen, Meng Li

**Affiliations:** a Archaeal Biology Center, Institute for Advanced Study, Shenzhen University, Shenzhen, China; b Key Laboratory of Optoelectronic Devices and Systems, College of Physics and Optoelectronic Engineering, Shenzhen University, Shenzhen, China; c Key Laboratory of Marine Ecosystem Dynamics, Second Institute of Oceanography, Ministry of Natural Resources, Hangzhou, China; d Department of Ocean Science and Hong Kong Branch of Southern Marine Science and Engineering Guangdong Laboratory (Guangzhou), The Hong Kong University of Science and Technology, Hong Kong SAR, China; e Shenzhen Key Laboratory of Marine Microbiome Engineering, Institute for Advanced Study, Shenzhen University, Shenzhen, Guangdong, China; f Southern Marine Science and Engineering Guangdong Laboratory (Zhuhai), Zhuhai, China; University of Michigan-Ann Arbor

**Keywords:** *Thaumarchaeota*, *Bathyarchaeota*, hypoxia, distribution

## Abstract

Water hypoxia (DO < 2 mg/L) is a growing global environmental concern that has the potential to significantly influence not only the aquatic ecosystem but also the benthic sedimentary ecosystem. The Changjiang River Estuary hypoxia, classified as one of the world's largest seasonal hypoxic water basins, has been reported to be expanding rapidly in recent decades. However, the microbial community dynamics and responses to this water hypoxia are still unclear. In this study, we examined the abundance, community composition, and distribution of sedimentary archaea, one important component of microbial communities in the Changjiang River Estuary and the East China Sea (ECS). Our results indicated that *Thaumarchaeota* and *Bathyarchaeota* were predominant archaeal groups in these research areas, with their 16S rRNA gene abundance ranged from 8.55 × 10^6^ to 7.51 × 10^8^ and 3.18 × 10^5^ to 1.11 × 10^8^ copies/g, respectively. The sedimentary archaeal community was mainly influenced by DO, together with the concentration of ammonium, nitrate, and sulfide. In addition, distinct differences in the archaeal community's composition, abundance, and driving factors were discovered between samples from hypoxia and non-hypoxia stations. Furtherly, microbial networks suggest various microbes leading the different activities in hypoxic and normoxic environments. *Bathyarchaeota* and *Thermoprofundales* were “key stone” archaeal members of the low-DO network, whereas *Thaumarchaeota* constituted a significant component of the high-DO network. Our results provide a clear picture of the sedimentary archaeal community in coastal hypoxia zones and indicates potential distinctions of archaea in hypoxia and non-hypoxia environments, including ecological niches and metabolic functions.

**IMPORTANCE** In this study, the sedimentary archaeal community composition and abundance were detailed revealed and quantified based on 16S rRNA genes off the Changjiang River Estuary. We found that the community composition was distinct between hypoxia and non-hypoxia regions, while *Thaumarchaeota* and *Bathyarchaeota* dominated in non-hypoxia and hypoxia samples, respectively. In hypoxia regions, the sedimentary archaea were mainly affected by salinity, ammonium, and nitrate, whereas total organic carbon, total nitrogen, and sulfide were major influencing factors in non-hypoxia regions. The distinct microbial network may suggest the niche difference of archaeal community under various oxygen level.

## INTRODUCTION

Because archaea were proposed as the third domain of life by Carl Woese (1, 2), they have been observed in a wide variety of habitats, encompassing both terrestrial and marine ecosystems (3–5). Although only few archaeal species are cultured to date, the cultivation-independent techniques, including metagenomics and single-cell genomics have offered a more comprehensive picture of archaea diversity, metabolic potential, and ecological significance (6, 7). For example, two archaeal phyla, *Thaumarchaeota* and *Bathyarchaeota*, are repeatedly detected in estuarine and coastal surface sediments, where they play a significant role in the sedimentary nitrogen and carbon cycles, respectively (8–11). Marine sediments are one of the greatest reservoirs of prokaryotic cells (5, 12). Notably, archaea are estimated to constitute approximately 40 and 12.8% of prokaryotic cells, respectively, in sediments of marginal regions and open ocean sites (13), highlighting their potential importance in these regions.

Marine Group I archaea (MG-I) are regarded to be the most abundant and widespread *Thaumarchaeota* in marine and coastal environments (3, 14, 15). Nitrosopumilus maritimus SCM1 (SCM1), one typical species of MG-I, is the first isolate of ammonia oxidizing archaea (16). Subsequent studies have revealed the widespread distribution of this ammonia oxidizing archaea (AOA) based on *amoA* (the gene coding for the α-subunit of the ammonia monooxygenase of ammonia oxidation) genes survey, thus highlighting their critical role in worldwide nitrification processes (8, 17). *Bathyarchaeota* (Miscellaneous Crenarchaeotal Group, MCG), as a cosmopolitan archaeal lineage with high degree of phylogenetic diversity, are found abundant in sediments ranging from organic-rich coastal locations to pelagic oceans (9, 18–20). Genomic evidence suggests that members of *Bathyarchaeota* might utilize a diverse range of organic molecules heterotrophically, including as detrital proteins, aromatic compounds, and plant-derived carbohydrates (18, 19). Based on genomic studies, *Bathyarchaeota* members have recently been proposed to have metabolic capacities for methanogenesis and acetogenesis (21–23), highlighting their potential contribution to benthic carbon cycles. As a result, it is vital to conduct a thorough examination of both *Thaumarchaeota* and *Bathyarchaeota* to better understand their roles in biochemical cycles.

Dissolved oxygen (DO) concentrations in natural waters, which are regulated by oxygen supply and removal processes, are critical for regulating nutrient fluxes, driving biotic interactions, and shaping microbial communities in aquatic ecosystems. Previous studies have explored the long-term hypoxia in bottom-water layers of the East China Sea (ECS) (24–26). Coastal hypoxia can occur naturally as a result of upwellings, algae blooms, or natural occurrences (e.g., storms), which are more prevalent in still basins, fjords, and estuaries (27, 28). However, more frequently, anthropogenic impacts appear to have a significant effect on coastal hypoxia, for example, eutrophication (29, 30). In addition, global warming may decrease oxygen solubility and decrease water column ventilation, resulting in oxygen minimum zones enlargement (31, 32).

The marginal zone of the ECS is confronted with numerous environmental problems, including terrigenous anthropogenic inputs and eutrophication (33, 34), heavy metal pollution (35), and water hypoxia (36). Water column hypoxia persists and becomes severe in summer off the Changjiang River Estuary (CRE) and ECS, primarily due to eutrophication (37, 38). According to previous research, various archaeal groups were observed in the estuarine surface sediments (39–42). However, their distribution and abundance in hypoxic zones have been mostly unknown, particularly for *Thaumarchaeota* and *Bathyarchaeota*. The purpose of this study was to determine the composition and quantity of the archaeal community in surface sediments of the ECS off the CRE mainly based on the 16S rRNA genes, focusing on *Thaumarchaeota* and *Bathyarchaeota*. The high-throughput sequencing and cultivation-independent techniques have greatly broadened the understanding of archaeal diversity and distribution, including the usage of 16S rRNA genes. However, technical biases, such as sampling methods, library construction approaches, and different usage of primers, might bring uncertainty on the microbial community composition. Although it is difficult to measure the contribution of all variables, a precise and lucid understanding relies on considering different aspects as much as possible. In this study, the archaeal community differed significantly across hypoxic and normoxic zones, and DO was a significant driving force influencing the distribution pattern of *Thaumarchaeota* and *Bathyarchaeota*. Our findings provide a more comprehensive picture of the archaeal community in hypoxia sediments and propose possible explanations for their ecological roles in hypoxia habitats.

## RESULTS

### Characteristics of archaeal community in samples.

**FIG 1:**
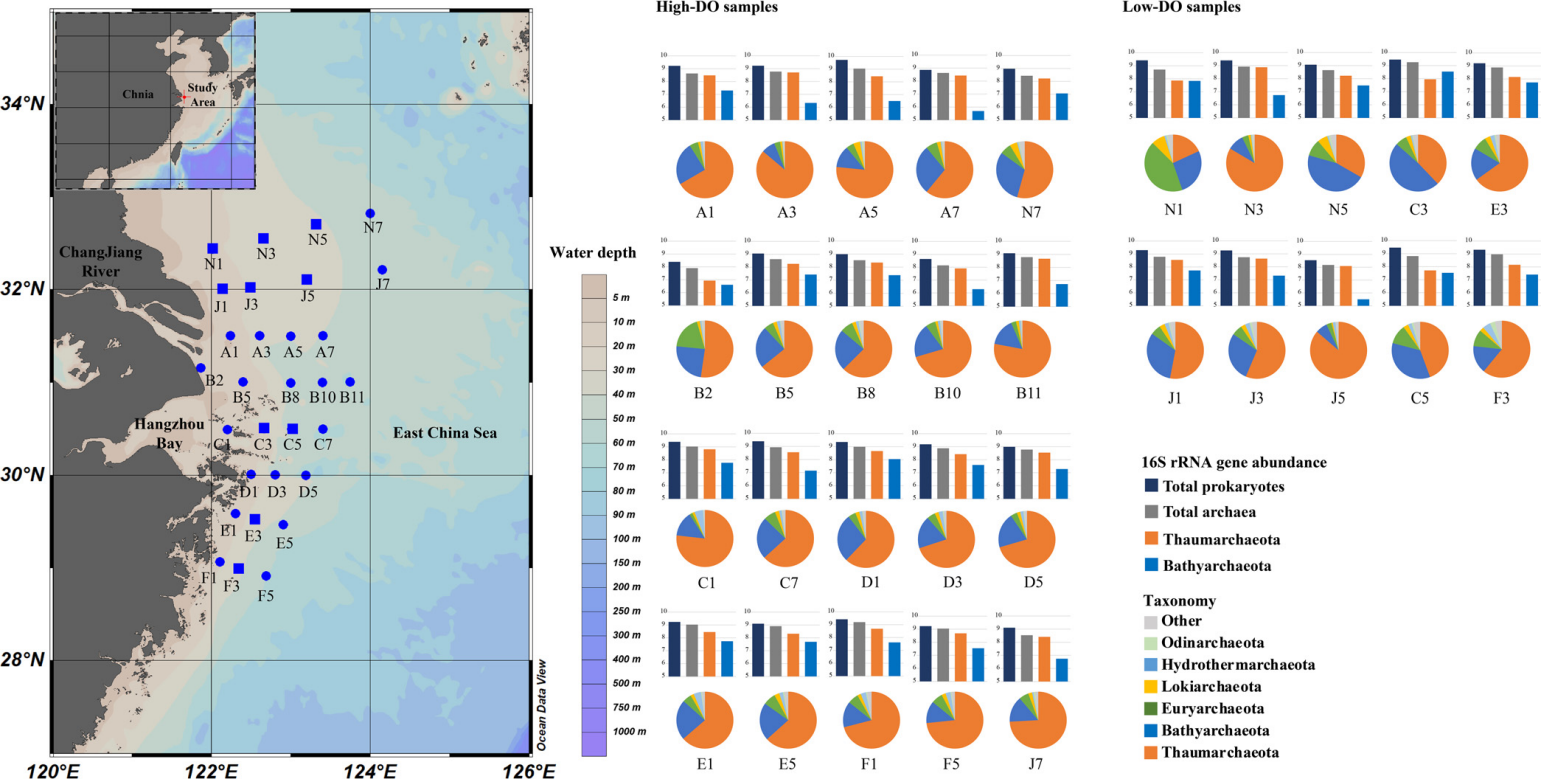
Location, the archaeal community composition (in phylum level), and the abundance of 16S rRNA genes in different categories of samples. Square stands for low-DO samples, solid circle stands for high-DO samples. The map was generated with Ocean Data View software.

DO saturation in bottom-water layers ranged from 1.13 to 104.63%, establishing three hypoxic areas in the ECS from north to south, comprising stations N1, N3, N5, J1, J3, J5, C3, C5, E3, and F3 (Table S1 in the supplemental material; Fig. 1). Besides, the NO_3_^−^, NO_2_^−^, and NH_4_^+^ concentrations were highest at stations B2, A3, and N1, respectively. Total organic carbon (TOC) and total nitrogen (TN) concentrations were highest near the coast, most likely due to anthropogenic inputs from the land, and gradually reduced in the outer regions.

Archaeal read numbers ranged from 32,702 (at station J5) to 58,647 (at station A1) in the 30 samples (Table S3 in the supplemental material). The rarefaction curve suggests that the depth of the sequencing is sufficient (Fig. S3). Table S3 presents the diversity index for each sample, including the observed operational taxonomic unit (OTU) counts, as well as the Shannon and Simpson indices. This investigation identified 3,350 archaeal OTUs in total, with the lowest number being 145 at station J5. Notably, the archaeal community diversity index does not differ significantly across the low- and high-DO samples (Fig. S4).

The composition of the archaeal community at the phylum level for each sample is depicted in Fig. 1. *Thaumarchaeota* and *Bathyarchaeota* were the most abundant archaeal taxa in the surface sediments, accounting for approximately 63.3 and 22.2% of all archaeal readings, respectively (Table S4 in the supplemental material). Specifically, the majority of thaumarcheotal sequences belonged to *Ca.* Nitrosopumilus, which was classified into three groups (i.e., N1, N2, and N3) (Fig. S5). Although Bathy-15, -17, -1, -8, and -6 were major subgroups of *Bathyarchaeota*, accounting for > 90% proportion of the bathyarchaeotal community (Fig. S4). In addition, sequences of *Euryarchaeota*, *Hydrothermarchaeota*, *Lokiarchaeota*, and *Odinarchaeota* were also identified. Low- and high-DO samples were clustered together respectively based on archaeal community composition, and the proportion of *Thaumarchaeota*, *Bathyarchaeota*, *Euryarchaeota*, *Lokiarchaeota*, and *Odinarchaeota* in the two groups was significantly different (*P < *0.05) (Fig. S6). Despite belonging to distinct DO groups, samples J5 and A3, as well as N7 and J1 had a similar community composition in phylum-level, while their detailed community composition was still quite different (Fig. S5). The core metabolic potentials of low- and high-DO samples were predicted and compared (Fig. S7). In terms of general metabolic of carbon, sulfur, and nitrogen, the differences were not obviously in different DO conditions. Although high-DO samples had higher fractions of metabolic potentials related to carbohydrates, amino acids, and nucleotides.

The total prokaryotes (bacteria and archaea) and total archaea were measured using 16S rRNA gene qPCR analysis (Table S3 in the supplemental material). The abundance of 16S rRNA gene of bacteria and archaea ranged from 2.44 × 10^8^ to 5.31 × 10^9^ copies/g. The abundance of archaeal 16S rRNA gene ranged from 7.95 × 10^7^ to 1.81 × 10^9^ copies/g. Besides, the abundance of 16S rRNA gene of *Thaumarchaeota* and *Bathyarchaeota* was also quantified via qPCR analysis with specific primers (Table S3), with the abundance range from 8.55 × 10^6^ to 7.51 × 10^8^ copies/g, and from 3.18 × 10^5^ to 1.11 × 10^8^ copies/g, respectively. There were no significant variations in the abundance of total prokaryotes and archaea between the low- and high-DO groups (Fig. S4). However, the 16S rRNA gene abundance of *Thaumarchaeota* was significantly higher (*P < *0.05) in high-DO samples than in low-DO samples, while *Bathyarchaeota* were significantly more abundant (*P < *0.05) in low-DO samples than in high-DO samples (Fig. S4). The results corroborate previous findings that *Thaumarchaeota* and *Bathyarchaeota* were enriched in samples with high and low DO, respectively (Fig. S5).

**FIG 2:**
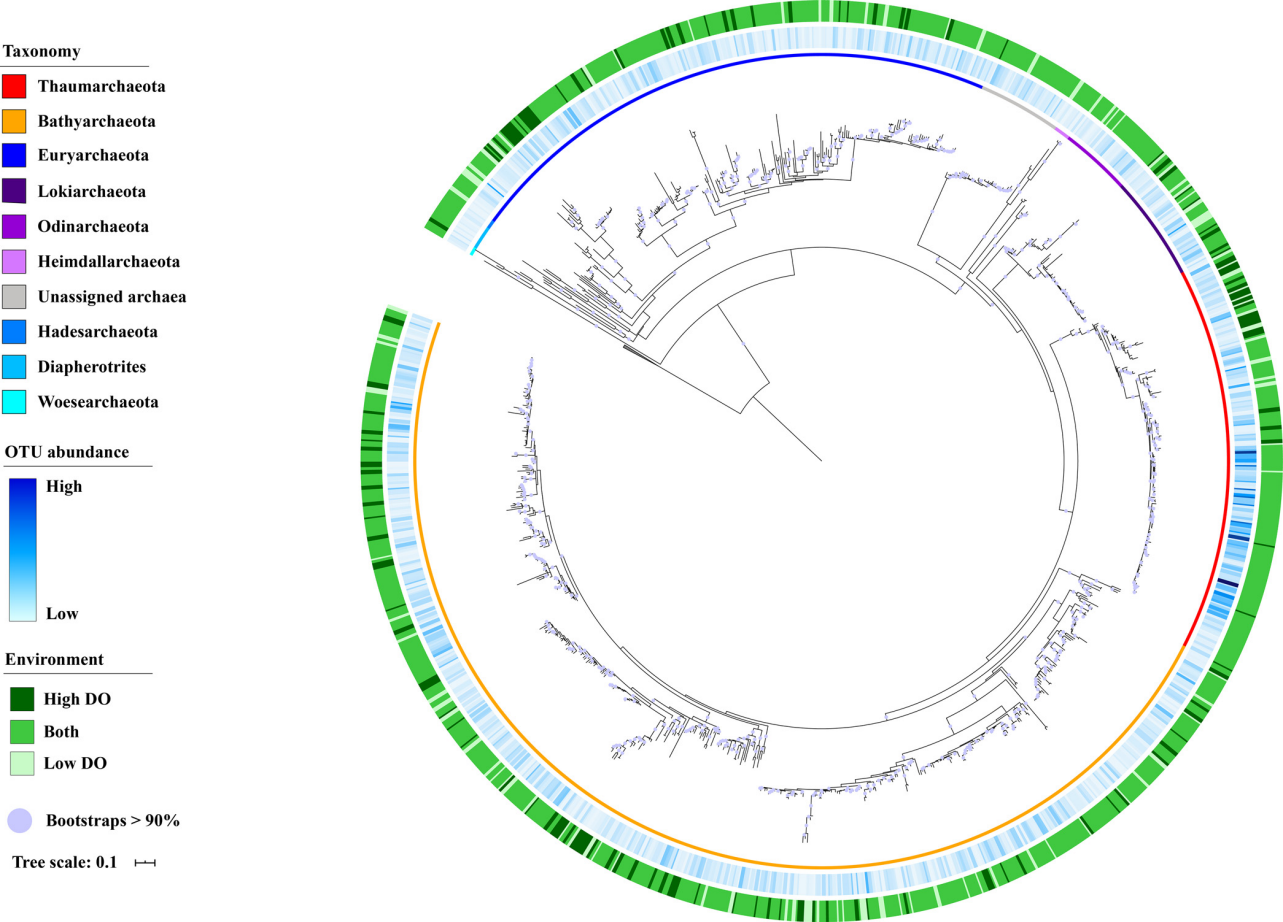
The phylogenetic tree of representative archaeal OTUs. The inner ring stands for the taxonomy information. The middle ring stands for the average relative abundance of each OTU in samples. The outer ring shows that the OTU was only observed in low- (light green) and high-DO (dark green) samples or was found in both sample groups (green).

The phylogenetic tree depicts the phylogeny of representative archaeal OTUs (Fig. 2). *Bathyarchaeota* accounted for roughly half of the representative OTUs, with a total of 20 subgroups (Fig. S5 in the supplemental material). Thaumarchaeotal OTUs were more abundance than others, and the majority were observed in both low- and high-DO samples, representing the study's key components. Nevertheless, the DO demand for bathyarchaeotal OTUs was so varied that several OTUs were only detected in low- or high-DO samples.

### Relationships between the community properties and physicochemical factors.

PCoA diagrams delineating the distinct community composition of archaea in low- and high-DO samples, with the first and second axis explained 33.22 and 17.76% of the variation, respectively (Fig. S8 in the supplemental material). The PCoA results showed that there were no distinct borders for samples with different DO. Similar patterns were observed for the thaumarchaeotal and bathyarchaeotal community. Besides, significant differences (*P < *0.05) were identified in terms of the community composition of *Thaumarchaeota* and *Bathyarchaeota* between the two groups.

**FIG 3:**
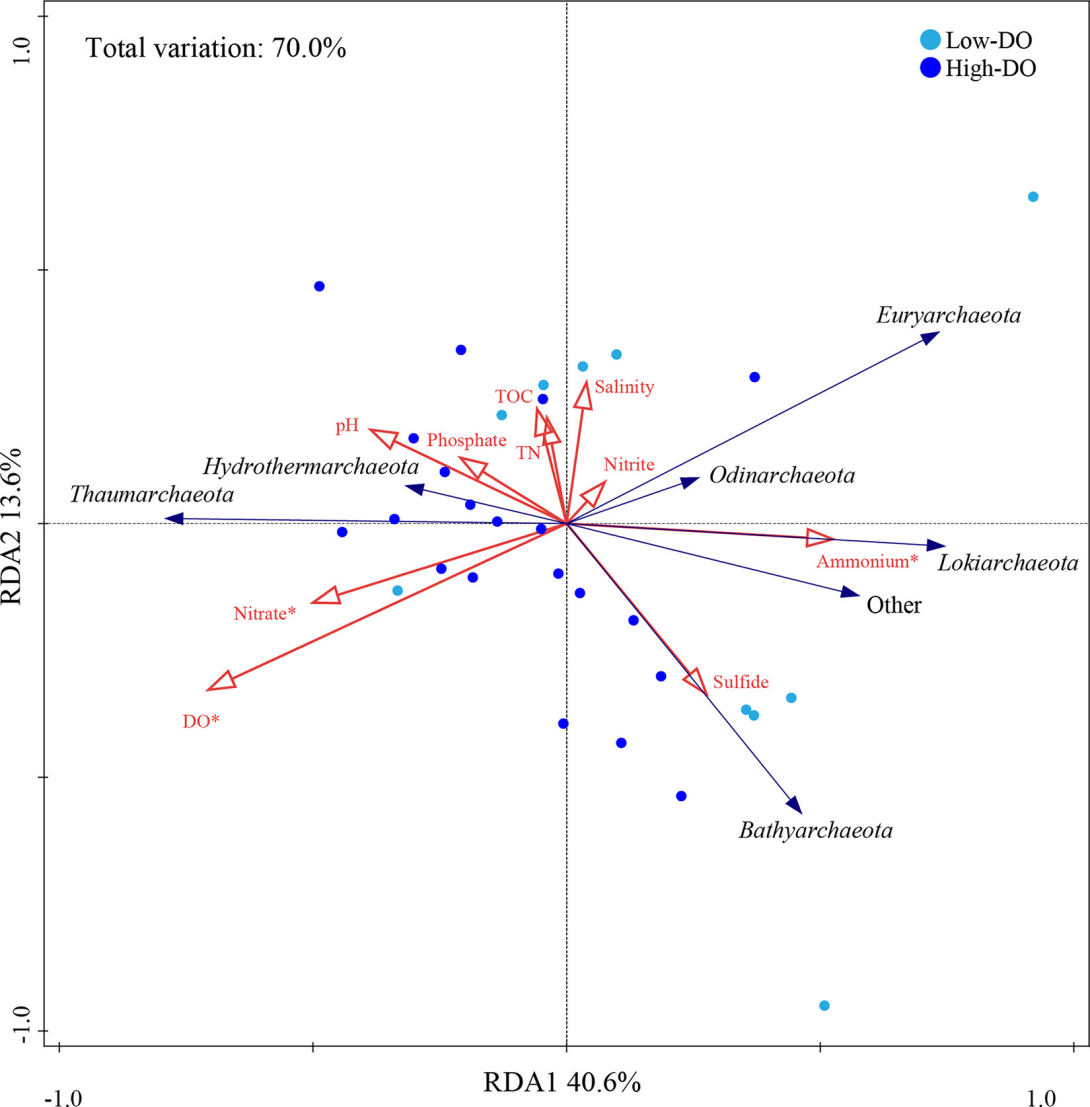
RDA results for the relationship between the environmental factors and the major archaeal phyla. Red arrows represent the factors, blue arrows represent the taxa. Dark-blue points represent the high-DO samples, and light-blue points represent the low-DO samples.

Further, the db-RDA demonstrated that DO, NH_4_^+^, NO_3_^−^, and sulfide were the most important contributing factors that influenced the archaeal community, explaining 23.8, 12.5, 11.6, and 10.3% (*P < *0.05) of total variation (70.0%), respectively (Fig. 3). Among the major archaeal phyla, *Thaumarchaeota* was closely associated with DO and NO_3_^−^, whereas *Bathyarchaeota* was mainly affected by sulfide. The NH_4_^+^ was the most influential factor in low-DO samples, explaining 23.7% (*P < *0.05) of the total community variation (Fig. S9a). Sulfide was the most influential factor in high-DO samples, explaining 37.2% (*P < *0.05) of the total community variation (Fig. S9b).

**FIG 4:**
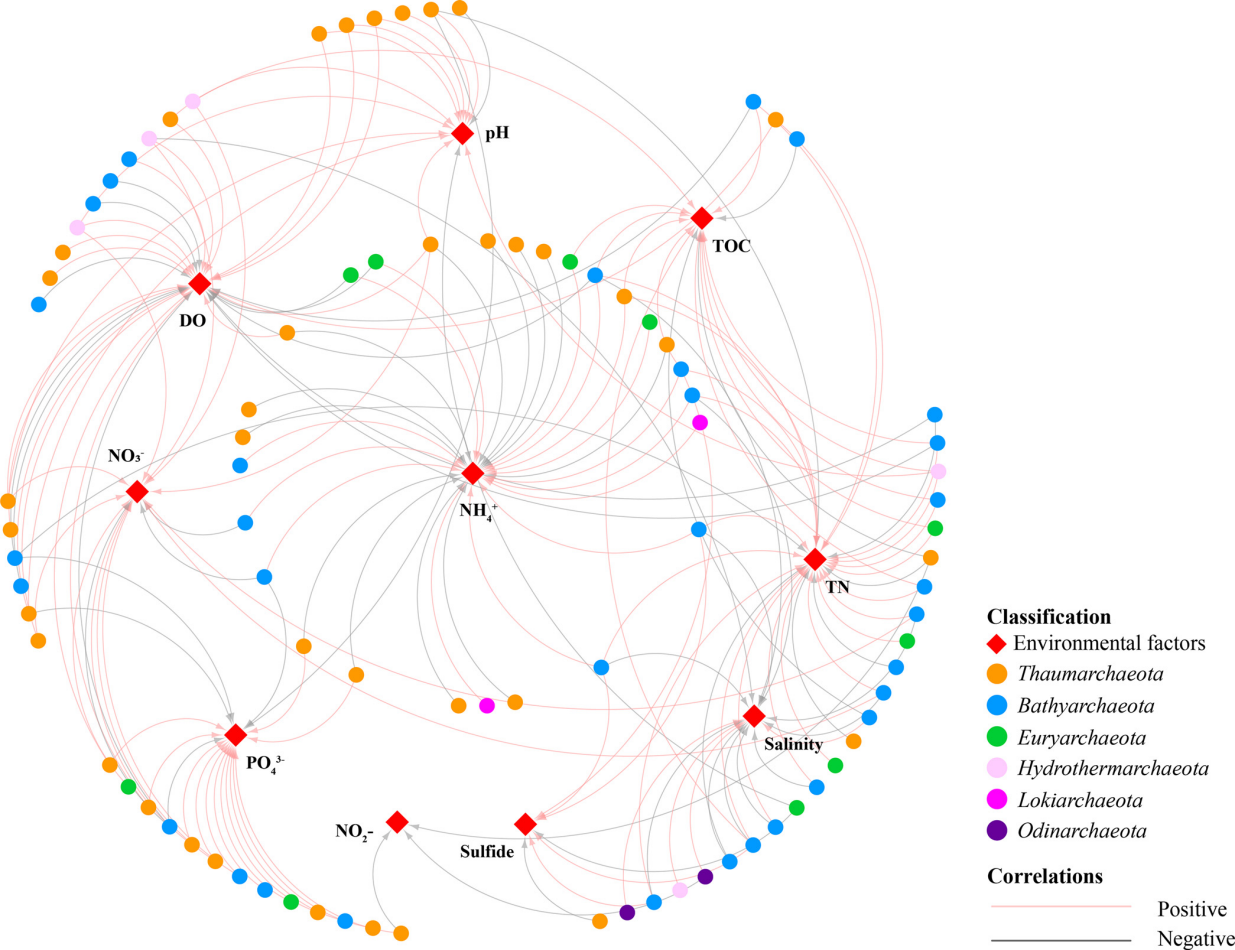
Relationships between the major archaeal OTUs and the environmental factors based on Pearson’s correlation coefficient. Only the |coefficient| > 0.7 with *P < *0.01 are showed in the network.

Pearson correlation analysis was employed to describe the relationship between the environmental factors and the archaeal community properties of the low- and high-DO sample groups (Fig. S10). Salinity, DO, and NO_3_^−^ were positively correlated with the diversity index in high-DO samples but showed opposite relationships in low-DO samples. TOC and sulfide had positive correlation to the abundance of *Bathyarchaeota* in high-DO samples but did not reveal strong correlations in low-DO samples. Besides, most subgroups of *Bathyarchaeota* were strongly affected by NH_4_^+^ (positive) and salinity (negative) (*P < *0.05) only in low-DO samples. Further, the influence of environmental factors on major archaeal OTUs was illustrated as well (Fig. 4). Most thaumarchaeotal OTUs were positively partitioned by DO, pH, and NO_3_^−^ yet negatively correlated with NH_4_^+^ (all *P < *0.05). As for Bathyarchaeota, they were mainly affected by DO, salinity, TOC, TN, and NH_4_^+^.

**FIG 5:**
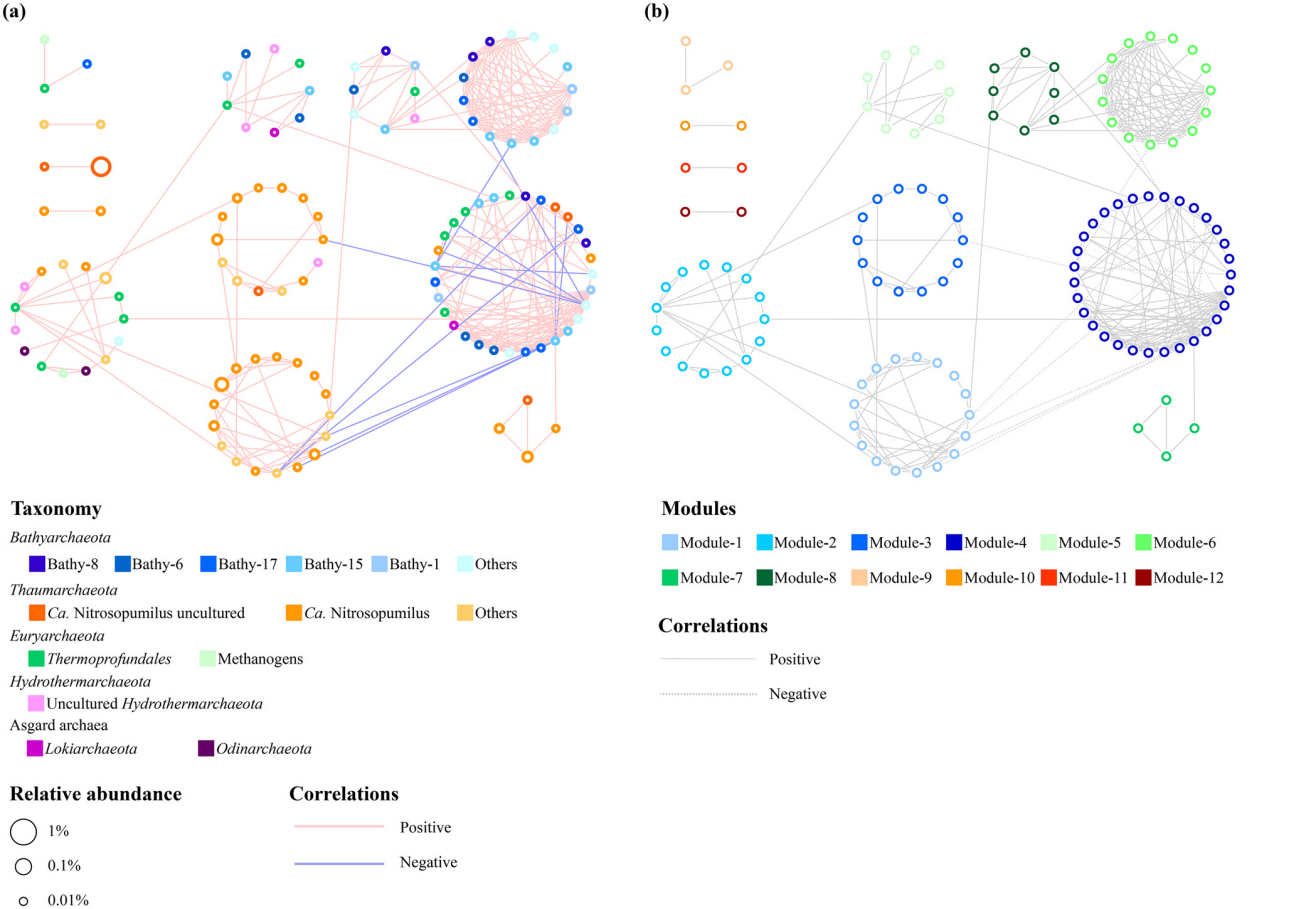
Microbial co-occurrence networks (a) and predicted module composition (b) of the archaeal community represented by major OTUs. Only OTUs observed in ≥15 samples, and interactions with *P < *0.01 are showed in the network.

The co-occurrence patterns of major archaeal groups are revealed by the network analysis (Fig. 5a). The network comprised of 122 nodes, representing the major archaeal OTUs, with 276 edges in total. Clearly, OTUs of *Thaumarchaeota* had relatively simple interactions, largely containing the positive associations with other thaumarchaeotal OTUs. Conversely, *Bathyarchaeota* revealed complicated interconnections. Except that they were negatively correlated with *Ca.* Nitrosopumilus or positively related to other *Bathyarchaeota*, members of *Bathyarchaeota* showed positive interactions with *Thermoprofundales* (Marine Benthic Group D [MBG-D]), *Hydrothermarchaeota*, and *Lokiarchaeota*. Further, the network is classified into 12 modules to designate the potential ecological niches (Fisher’s test, *P < *0.001) within the archaeal community in this study (Fig. 5b). *Thaumarchaeota* were observed in 8 modules, among which 5 modules solely constituted by *Thaumarchaeota*, whereas *Bathyarchaeota* were found in 6 modules with 1 module entirely consisting of them. Notably, the co-occurrence of *Bathyarchaeota* and *Thermoprofundales* were found in four modules showing a relatively close ecological connection. Besides, the interactions between *Bathyarchaeota*, *Hydrothermarchaeota*, and *Lokiarchaeota*, between *Thaumarchaeota* and *Hydrothermarchaeota*, and between *Thermoprofundales*, *Lokiarchaeota*, and methanogens, were displayed in more than one module.

Furthermore, possible microbial networks in low- and high-DO environments were also reconstructed (Fig. S11 in the supplemental material). The low-DO network featured more complicated interactions than the high-DO network, however they constituted less modules (8 modules) than the high-DO network (11 modules). Most interactions in the low-DO network were associated with *Bathyarchaeota* and *Thermoprofundales*, while they had less interactions in the high-DO network where *Thaumarchaeota* were discovered in practically all modules (9 out of 11 modules).

## DISCUSSION

### The archaeal community composition and differences in the low- and high-DO sediments.

According to a recent study, archaea cells formed a considerable part of microbial cells in marine sediments (approximately 37.3%), with a notably higher abundance in marginal areas (approximately 40.0%) than in open-ocean sites (approximately 12.8%) (13). Previous research indicates that archaea form a significant component of the microbiological population in the Changjiang River Estuary (CJE) and ECS surface sediments (39, 40, 42). Archaeal 16S rRNA gene accounted for 19.6–65.2% of the total prokaryotic community, wherea *Thaumarchaeota* (dominated by *Ca.* Nitrosopumilus) and *Bathyarchaeota* (dominated by Bathy-15, -17, -1, -8, and -6) were prominent archaeal groups in the current study (Fig. S4 in the supplemental material). Seasonal variances of archaeal communities in sediments were reported in different habitats (43–45). A recent study revealed that the archaeal 16S rRNA gene was more abundant in winter than in summer of sediment samples from Chinese marginal regions, including the CJE and ECS, whereas MG-I and *Bathyarchaeota* dominated in both seasons (39). Besides, the more distinct intra-area variation in archaeal abundance in summer than in winter, especially for estuarine samples, which may indicate the niche partitioning within a small spatial scale in summer (39). Hence, it is crucial to investigate and compare the abundance and distribution patterns of archaea in different seasons to better understand their ecological functions and niches.

The diversity index, the abundance of prokaryotic and archaeal 16S rRNA gene did not show significant differences between the low- and high-DO samples (Fig. S4 in the supplemental material). However, considerable changes in the composition of archaeal communities were detected. *Thaumarchaeota* 16S rRNA genes were abundant in high-DO samples, whereas *Bathyarchaeota* were on the opposite. This is because their lifestyles and oxygen requirements are distinct (17, 46). *Ca.* Nitrosopumilus are the most prevalent AOA found in a wide variety of environments (8, 11, 47, 48). They contribute significantly to aerobic ammonia oxidation and are involved in a wide variety of biochemical cycles (16, 49, 50). Different AOA species or ecotypes may have varying oxygen tolerance ranges (51), and the low-oxygen and micro-aerobic condition may benefit the growth of certain members. *Bathyarchaeota* are reported to have potential anaerobic lifestyles in sediments with the ability of degrading organic compounds, such as carbohydrates and proteins (9, 18, 52). Apart from heterotrophic degradations, most *Bathyarchaeota* may gain energy via the anaerobic Wood–Ljungdah pathway, and certain individuals encode genes related to the Calvin-Benson-Bassham cycle (23, 53). As a result, they were more abundant in the low-DO samples than high-DO samples in this study.

The OTU numbers of *Thaumarchaeota* and *Bathyarchaeota* were significantly greater (*P < *0.05) in high- than low-DO samples. This may indicate a high diversity of them in high-DO environments. While the majority of *Thaumarchaeota* and *Bathyarchaeota* OTUs were recognized in both sample groups, many were found to reside in low- or high-DO samples, implying that they have a distinct ecological niche and adaptations for each DO state. Besides, the differences of archaeal community composition were also apparently illustrated in the sample clustering result and the PCoA plot. Therefore, it is vital to elucidate the mechanisms that contribute to the archaeal community distribution patterns in low- and high-DO samples, as well as to emphasize the potential influence of environmental factors.

### Driven factors of the archaeal distribution in low- and high-DO environments.

Bottom-water hypoxia may have an effect on the physicochemical properties of surface sediments, modify biogeochemical processes, and consequently, on benthic communities (28, 54). As indicated earlier, DO was a significant environmental factor dividing the archaeal community, impacting the abundance and diversity of *Thaumarchaeota* and *Bathyarchaeota* in the surface sediments. Several studies revealed that DO may be one of the key factors influencing microbial community spatial structure, also regulating nitrification and denitrification potentials in the CRE (41, 55, 56). The habitat expansion of AOA from terrestrial to marine environments may be mainly driven by oxygen (57). Besides, AOA was thought as major ammonia oxidizers in sediments of the CRE (56, 58). Recent reports indicated the metabolic potentials of estuarine AOA in utilizing carbohydrates (59, 60). The high abundance of *Thaumarchaeota* (mainly consisted of *Ca.* Nitrosopumilus) observed in the high-DO samples may highlight their contributions in the benthic nutrient cycles.

The concentrations of NH_4_^+^, NO_3_^−^, and sulfide also shaped the archaeal community (Fig. 3). Ammonium is an indispensable component of microbial communities because it serves as a critical substrate in the nitrogen cycle. According to metagenomics, members of the Bathy-6, -8, -15, and -17 encode genes associated to ammonium transporters, and may produce urea by utilizing diverse nitrogen compounds (23), which may be responsible to the positively correlation with ammonium. Similar patterns between *Bathyarchaeota* and ammonium were also reported in other estuarine (61), mangrove (62), and karstic limnic sediments (63). Certain AOA OTUs showed negative correlations to ammonium, possibly because to their predilection for low-ammonium environments and high-affinity of ammonium (64). In addition, some OTUs belonging to the *Thermoprofundales*, *Methanosarcinales*, and *Lokiarchaeota* showed positive correlations (*P < *0.05) with the ammonium concentration, as reported and explained in a recent study in the Pearl River Estuary (61).

Members of *Bathyarchaeota* (65), *Thermoprofundales* (66), and *Hydrothermarchaeota* (67) all encode *nar* (nitrate reductase) genes. This may account for the positive association identified in this study between nitrate and them (Fig. 4). Besides, they showed a positive correlation with TOC and TN, which may be explained by their predicted metabolic capability for decomposing organic molecules. Further, the concentration of TOC and TN was positively correlated with the 16S rRNA gene abundance of *Bathyarchaeota* (Fig. S10 in the supplemental material), which was consistent with earlier studies (62, 68). *Bathyarchaeota* and *Hydrothermarchaeota* are reportedly to participate in a variety of sulfur metabolisms (23, 67), which may explain their tight association with sulfide. Other variables, such as salinity, were primarily associated with *Bathyarchaeota*, whereas pH and phosphate were primarily associated with *Thaumarchaeota*. These findings emphasize the critical function of archaea in sedimentary nitrogen cycling and shed light on the ecological niches of various archaeal groups.

### Potential microbial interactions within the archaeal community.

Liu et al. (39) observed clear spatiotemporal patterns of the sedimentary archaeal community structure while stochasticity had a greater role than determinism in structuring this distance-decay pattern. However, their results also indicated that the archaeal co-occurrence relationships changed over seasons, with closer inter-taxa connections observed in winter than in summer. Their findings may suggest highly complex dynamics of sedimentary archaeal communities and inter-taxa interactions. In the current study, within *Bathyarchaeota*, interactions between OTUs from various subgroups were extremely complicated, showing high diversity and niche variations. Conversely, the relatively simple interactions among *Ca*. Nitrosopumilus may be consistent with their unitary ammonia oxidation metabolic capacity.

*Bathyarchaeota* are believed to be capable of decomposing a wide variety of organic molecules, including carbohydrates, fatty acids, aromatic compounds, and proteins, as well as urea, acetate, methane, and methylation compounds (20, 22, 65, 69). *Bathyarchaeota* various metabolic capabilities underscore their critical role in benthic carbon cycles. Metagenomics indicates that the majority of *Bathyarchaeota* members are capable of acetogenesis and fermentation (9, 21, 53). Metagenomic analysis also illustrated variations in metabolic capacities, substrate preferences, and ecological niches of bathyarchaeotal subgroups (18, 21, 23), underlining their metabolic flexibility. *Thermoprofundales* is another important sedimentary archaeal group ubiquitously distributed in diverse ecosystems (5, 66). Similarly, based on genomic inferences, *Thermoprofundales* may be capable of exogenous protein mineralization, acetate and ethanol generation via fermentation, also fixing CO_2_ through the Wood–Ljungdahl pathway (20, 65, 66). As a result, they may act as substrates for heterotrophic microorganisms and acetoclastic methanogens in sediments, as well as assist carbon transformation in the ecosystem. *Thermoprofundales* interacted closely with methanogens (including *Methanofastidiosales* and *Methanosarcinales*) in the current investigation, which may imply a syntrophic connection for them. It is suggested that members of the *Hydrothermarchaeota* and *Lokiarchaeota* encode genes involved in acetate or ethanol fermentations (67). Besides, *Bathyarchaeota* and *Thermoprofundales* share similar metabolic pathways through genomic comparisons (66). This may account for the tight contacts identified in this study, indicating probable symbiotic or synergistic relationships among *Bathyarchaeota*, *Thermoprofundales*, *Hydrothermarchaeota*, and *Lokiarchaeota*. The high abundance and diverse metabolic potentials of *Bathyarchaeota* and *Thermoprofundales* may imply their significance in benthic biogeochemical cycling, especially in the low-DO regions.

Intriguingly, in the low-DO samples, the distribution of archaeal community was mainly influenced by NH_4_^+^, NO_3_^−^, and salinity, whereas sulfide, TN, and TOC were the most important driven factor in high-DO samples (Fig. S9 in the supplemental material). This could indicate that microbial activities differed between low- and high-DO settings, as well as the community composition. NO_3_^−^ and NH_4_^+^ were frequently used by microbes in reductions and anaerobic metabolisms in dysoxic or anoxic environments, whereas oxidation of organic molecules may be prevalent in microbial communities with an adequate supply of oxygen. Additionally, microbial interactions varied according on DO level (Fig. S11). Denitrifying microorganisms and anammox bacteria were previously reported to be significant contributors in the ECS hypoxic region's surface sediments, contributing to benthic nitrogen cycle (26, 70). Although the capacities of *Bathyarchaeota* and *Thermoprofundales* for denitrification and dissimilatory NO_3_^−^ reduction to NH_4_^+^ are still theoretically inferred via metagenomic analysis, their close relationship with NO_3_^−^ and NH_4_^+^ may shed light on their importance in the nitrogen cycle and provide possible clues for future explorations.

### Conclusions.

In summary, this study examined the abundance and community composition of sedimentary archaea in hypoxic and normoxic sections off the CRE and the ECS. Except for DO, the concentration of ammonium, nitrate, and sulfide were identified as significant factors affecting archaea abundance, diversity, and distribution. There were apparent variations in the archaeal community between hypoxia and non-hypoxia locations. *Thaumarchaeota* and *Bathyarchaeota* were found to be enriched in samples with high and low DO, respectively. In addition, the key driving variables were different in hypoxic and normoxic conditions, which may have resulted in the initiation of distinct metabolic processes. In addition, possible microbial interactions were different in low- and high-DO samples, indicating a niche difference in the archaeal community under differing oxygen levels.

## MATERIALS AND METHODS

### Study area, sampling, and physicochemical measurements.

The study region is located in the coastal ECS off the CRE and Hangzhou Bay, with latitudes ranging from 121.87° E to 124.15° E and longitudes ranging from 28.92° N to 32.82° N, totaling 30 stations (Fig. 1). Surface sediments of each sampling station were collected during a cruise in 13–26 August 2016. The sediments were sealed in 50 mL tubes immediately after grabbed from the water bed, stored in liquid nitrogen on board, and then transferred to a −40°C fridge in the laboratory before further analysis. Environmental factors of the bottom water above sediments of each site, including salinity, pH, DO, and the concentration of ammonium, nitrite, nitrate, and phosphate were measured. Briefly, water salinity, pH, and DO saturation were measured by the CTD (Sea-bird, America) on board. The concentrations of nutrients (NO_3_^–^, NO_2_^–^, NH_4_^+^, and PO_4_^3–^) were determined using an auto-analyzer (QuAAtro, Germany) in library. Besides, the concentration of TOC and TN were analyzed using TOC-LCPH/CPN (Shimadzu, Japan), whereas sulfide was determined using a silver-sulfide electrode (Thermo Scientific, America). All physicochemical factors were listed in Table S1 in the supplemental material.

### DNA extraction, sequencing, and data processing.

The PowerMax soil kit (Qiagen) was used according to the manufacturer's instructions to extract DNA from 10 g of wet sediments in triplicate for each sample. Triplicates of each sample were combined and well mixed prior to sequencing and analysis. The archaeal 16S rRNA gene amplification was performed using the primer pair Arch524F/Arch958R (F: 5′-TGYCAGCCGCCGGTAA-3′ and R: 5′-YCCGGCGTTGAVTCCAATT-3′), as previously described (61). PCR-free libraries were constructed from the 16S rRNA gene amplicons and sequenced using Illumina HiSeq2500 (USA) PE250 by Novogene (China) according to standard protocols.

The QIIME2 pipeline (version 2019.1) was used to analyze the sequence data, which included quality filtration, demultiplexing, denoising with dada2, taxonomy assignment, and phylogenetic and diversity analysis (71, 72). Taxonomic assignment of the representative archaeal sequences was achieved using the SILVA database (release 132) (73). Besides, based on recent studies, the detailed subgroup compositions were categorized of *Bathyarchaeota* (9) and *Thermoprofundales* (66). The OTU table was normalized by setting the uniform sequence number to 32702 for each sample to generate the alpha diversity index (i.e., the Shannon index, Simpson index, and the number of observed OTUs) of archaeal community and the Bray-Curtis dissimilarity between samples. Tax4Fun2 (version 1.1.14) was used to predict the functional profiles of low- and high-DO samples, and calculated the metabolic potential by linking the 16S rRNA gene abundance profile to Kyoto Encyclopedia of Genes and Genomes (KEGG) Orthology (KO) database (74).

### Quantitative PCR Analysis.

The 16S rRNA gene copy numbers of total prokaryotes (bacteria and archaea), total archaea, *Thaumarchaeota*, and *Bathyarchaeota* were quantified using real-time quantitative PCR (qPCR) via the QuantStudio3 instrument (Thermo Fisher Scientific) with standard protocols, and the primer pairs 515F/806R (75), Arch519F/Arch908R (76, 77), Thaum494/958R (78, 79), and MCG242dF/Bathy442R (62) were used respectively (Table S2 in the supplemental material). For the analysis, a 20-mL qPCR was prepared, containing the following: 10 mL of PowerUp SYBR green master mix (Applied Biosystems), 2 mL of DNA template, 1 mL each of the forward and reverse primers (10 mM), and 6 mL of ddH_2_O. The standard qPCR curves were generated using sequential 10-fold dilution series of the pMD19-T vector, and detailed methods to calculate the gene copy number of each qPCR experiment were based on a recent study (61). Gel electrophoretograms and melt curves were presented to verify the results of all qPCR experiments (Fig. S1 and S2).

### Statistical analysis.

Samples were categorized in two groups based on the DO value of each sample. In water columns of estuarine and coastal regions, DO < 2 mg/L (DO saturation < 25%) is usually defined as hypoxia (37, 38, 80). Thus, in the current study, samples with DO% < 25% were the low-DO group and others were the high-DO group. Besides, archaeal OTUs with average abundance fraction > 0.01% (776 OTUs in total, representing > 93.3% sequences) were selected for further analysis. The phylogenetic tree of representative was constructed using IQ-TREE (version 1.6.12) (81) and visualized in the online platform iTOL (version 4) (82). Sample clustering was employed using R (version 3.5.0) at archaeal OTU level and the unweighted pair-group method with arithmetic means (UPGMA). Analysis of similarities (ANOSIM) and the *t* test were implemented using IBM SPSS Statistics (version 20.0) without outliers to test the significance of differences in archaeal community between the low- and high-DO group, including the community composition, the diversity index, and the abundance of 16S rRNA genes. Principal coordinate analysis (PCoA) was employed to delineate the dissimilar relationship between samples based on the OTU composition for the total archaeal community, *Thaumarchaeota*, and *Bathyarchaeota*. Variance inflation factor (VIF) was used to verify the linearity relationship between all environmental factors, and to selected non-linear factors for further analysis (i.e., TOC, TN, sulfide, salinity, pH, NO_3_^−^, NO_2_^−^, NH_4_^+^, and PO_4_^3^^−^). The result of detrended correspondence analysis (DCA) suggested that redundancy analysis (RDA) was better to depict the influence of environmental factors on the ordination of samples and the compositional archaeal taxa based on the Bray-Curtis’s distance (db-RDA). Variation partitioning analysis (VPA) was employed to quantify the most contributing factors by RDA ordination. The analysis and visualization of PCoA, VIF, RDA, and VPA results were conducted via the vegan package (version 2.4–3) in R (version 3.5.0).

The normality of physicochemical parameters was examined using the Kolmogorov–Smirnov test. As all environmental factors were normal distribution, Pearson correlation analysis was used to describe the correlative relationship between environmental factors and the relative abundance, quantity, and diversity index in the current study through IBM SPSS Statistics (version 20.0). Besides, the Pearson correlation between the abundance of major OTUs (average abundance fraction > 0.05%, 217 OTUs in total, representing > 80.7% sequences) and environmental factors was illustrated by a network via Cytoscape (version 3.7.2). To depict the co-occurrence of different archaeal groups and predict potential microbial interactions, major OTUs were selected in network construction based on Pearson correlation using MENA online platform (http://ieg4.rccc.ou.edu/mena), and only edges with the |coefficient| > 0.7 and *P < *0.01, were retained and visualized through Cytoscape (version 3.7.2).

### Data availability.

The raw HiSeq sequencing data for 30 archaeal 16S rRNA gene libraries in this study were deposited in the National Omics Data Encyclopedia (NODE) database with the BioProject accession number OEP001115 and the BioSample accession number from OES045981 to OES046010.

## References

[1] Woese CR, Fox GE. 1977. Phylogenetic structure of the prokaryotic domain: the primary kingdoms. Proc Natl Acad Sci USA 74:5088–5090. doi:10.1073/pnas.74.11.5088. 270744 PMC432104

[2] Woese CR, Kandler O, Wheelis ML. 1990. Towards a natural system of organisms: proposal for the domains Archaea, Bacteria, and Eucarya. Proc Natl Acad Sci USA 87:4576–4579. doi:10.1073/pnas.87.12.4576. 2112744 PMC54159

[3] DeLong EF. 1992. Archaea in coastal marine environments. Proc Natl Acad Sci USA 89:5685–5689. doi:10.1073/pnas.89.12.5685. 1608980 PMC49357

[4] Bintrim SB, Donohue TJ, Handelsman J, Roberts GP, Goodman RM. 1997. Molecular phylogeny of Archaea from soil. Proc Natl Acad Sci USA 94:277–282. doi:10.1073/pnas.94.1.277. 8990199 PMC19314

[5] Biddle JF, Lipp JS, Lever MA, Lloyd KG, Sørensen KB, Anderson R, Fredricks HF, Elvert M, Kelly TJ, Schrag DP, Sogin ML, Brenchley JE, Teske A, House CH, Hinrichs K-U. 2006. Heterotrophic Archaea dominate sedimentary subsurface ecosystems off Peru. Proc Natl Acad Sci USA 103:3846–3851. doi:10.1073/pnas.0600035103. 16505362 PMC1533785

[6] Rinke C, Schwientek P, Sczyrba A, Ivanova NN, Anderson IJ, Cheng J-F, Darling A, Malfatti S, Swan BK, Gies EA, Dodsworth JA, Hedlund BP, Tsiamis G, Sievert SM, Liu W-T, Eisen JA, Hallam SJ, Kyrpides NC, Stepanauskas R, Rubin EM, Hugenholtz P, Woyke T. 2013. Insights into the phylogeny and coding potential of microbial dark matter. Nature 499:431–437. doi:10.1038/nature12352. 23851394

[7] Baker BJ, De Anda V, Seitz KW, Dombrowski N, Santoro AE, Lloyd KG. 2020. Diversity, ecology and evolution of Archaea. Nat Microbiol 5:887–900. doi:10.1038/s41564-020-0715-z. 32367054

[8] Francis CA, Roberts KJ, Beman JM, Santoro AE, Oakley BB. 2005. Ubiquity and diversity of ammonia-oxidizing archaea in water columns and sediments of the ocean. Proc Natl Acad Sci USA 102:14683–14688. doi:10.1073/pnas.0506625102. 16186488 PMC1253578

[9] Zhou Z, Pan J, Wang F, Gu J-D, Li M. 2018. Bathyarchaeota: globally distributed metabolic generalists in anoxic environments. FEMS Microbiol Rev 42:639–655. doi:10.1093/femsre/fuy023. 29790926

[10] Liu X, Pan J, Liu Y, Li M, Gu J-D. 2018. Diversity and distribution of Archaea in global estuarine ecosystems. Science of the Total Environment 637–638:349–358. doi:10.1016/j.scitotenv.2018.05.016. 29753224

[11] Zou D, Liu H, Li M. 2020. Community, distribution, and ecological roles of estuarine Archaea. Front Microbiol 11:2060. doi:10.3389/fmicb.2020.02060. 32983044 PMC7484942

[12] Lipp JS, Morono Y, Inagaki F, Hinrichs K-U. 2008. Significant contribution of Archaea to extant biomass in marine subsurface sediments. Nature 454:991–994. doi:10.1038/nature07174. 18641632

[13] Hoshino T, Inagaki F. 2019. Abundance and distribution of Archaea in the subseafloor sedimentary biosphere. ISME J 13:227–231. doi:10.1038/s41396-018-0253-3. 30116037 PMC6298964

[14] Karner MB, DeLong EF, Karl DM. 2001. Archaeal dominance in the mesopelagic zone of the Pacific Ocean. Nature 409:507–510. doi:10.1038/35054051. 11206545

[15] Munson MA, Nedwell DB, Embley TM. 1997. Phylogenetic diversity of Archaea in sediment samples from a coastal salt marsh. Appl Environ Microbiol 63:4729–4733. doi:10.1128/aem.63.12.4729-4733.1997. 9406392 PMC168796

[16] Könneke M, Bernhard AE, José R, Walker CB, Waterbury JB, Stahl DA. 2005. Isolation of an autotrophic ammonia-oxidizing marine archaeon. Nature 437:543–546. doi:10.1038/nature03911. 16177789

[17] Hatzenpichler R. 2012. Diversity, physiology, and niche differentiation of ammonia-oxidizing archaea. Appl Environ Microbiol 78:7501–7510. doi:10.1128/AEM.01960-12. 22923400 PMC3485721

[18] Meng J, Xu J, Qin D, He Y, Xiao X, Wang F. 2014. Genetic and functional properties of uncultivated MCG archaea assessed by metagenome and gene expression analyses. ISME J 8:650–659. doi:10.1038/ismej.2013.174. 24108328 PMC3930316

[19] Kubo K, Lloyd KG, Biddle JF, Amann R, Teske A, Knittel K. 2012. Archaea of the Miscellaneous Crenarchaeotal Group are abundant, diverse and widespread in marine sediments. ISME J 6:1949–1965. doi:10.1038/ismej.2012.37. 22551871 PMC3449235

[20] Lloyd KG, Schreiber L, Petersen DG, Kjeldsen KU, Lever MA, Steen AD, Stepanauskas R, Richter M, Kleindienst S, Lenk S, Schramm A, Jørgensen BB. 2013. Predominant archaea in marine sediments degrade detrital proteins. Nature 496:215–218. doi:10.1038/nature12033. 23535597

[21] He Y, Li M, Perumal V, Feng X, Fang J, Xie J, Sievert S, Wang F. 2016. Genomic and enzymatic evidence for acetogenesis among multiple lineages of the archaeal phylum Bathyarchaeota widespread in marine sediments. Nat Microbiol 1:1–9. doi:10.1038/nmicrobiol.2016.35. 27572832

[22] Evans PN, Parks DH, Chadwick GL, Robbins SJ, Orphan VJ, Golding SD, Tyson GW. 2015. Methane metabolism in the archaeal phylum Bathyarchaeota revealed by genome-centric metagenomics. Science 350:434–438. doi:10.1126/science.aac7745. 26494757

[23] Pan J, Zhou Z, Béjà O, Cai M, Yang Y, Liu Y, Gu J-D, Li M. 2020. Genomic and transcriptomic evidence of light-sensing, porphyrin biosynthesis, Calvin-Benson-Bassham cycle, and urea production in Bathyarchaeota. Microbiome 8:1–12. doi:10.1186/s40168-020-00820-1. 32234071 PMC7110647

[24] Ye Q, Wu Y, Zhu Z, Wang X, Li Z, Zhang J. 2016. Bacterial diversity in the surface sediments of the hypoxic zone near the Changjiang Estuary and in the East China Sea. Microbiologyopen 5:323–339. doi:10.1002/mbo3.330. 26817579 PMC4831476

[25] Liu M, Xiao T, Wu Y, Zhou F, Huang H, Bao S, Zhang W. 2012. Temporal distribution of bacterial community structure in the Changjiang Estuary hypoxia area and the adjacent East China Sea. Environ Res Lett 7:e025001. doi:10.1088/1748-9326/7/2/025001.

[26] Wu D-M, Dai Q-P, Liu X-Z, Fan Y-P, Wang J-X. 2019. Comparison of bacterial community structure and potential functions in hypoxic and non-hypoxic zones of the Changjiang Estuary. PLoS One 14:e0217431. doi:10.1371/journal.pone.0217431. 31170168 PMC6553723

[27] Anderson JJ, Devol AH. 1987. Extent and intensity of the anoxic zone in basins and fjords. Deep Sea Res Part A Oceanographic Res Papers 34:927–944. doi:10.1016/0198-0149(87)90046-X.

[28] Middelburg J, Levin L. 2009. Coastal hypoxia and sediment biogeochemistry. Biogeosciences Discussions 6.

[29] Zhang J, Gilbert D, Gooday AJ, Levin L, Naqvi SWA, Middelburg JJ, Scranton M, Ekau W, Peña A, Dewitte B, Oguz T, Monteiro PMS, Urban E, Rabalais NN, Ittekkot V, Kemp WM, Ulloa O, Elmgren R, Escobar-Briones E, Van der Plas AK. 2010. Natural and human-induced hypoxia and consequences for coastal areas: synthesis and future development. Biogeosciences 7:1443–1467. doi:10.5194/bg-7-1443-2010.

[30] Rabalais N, Diaz RJ, Levin L, Turner R, Gilbert D, Zhang J. 2010. Dynamics and distribution of natural and human-caused hypoxia. Biogeosciences 7:585–619. doi:10.5194/bg-7-585-2010.

[31] Bendtsen J, Hansen JL. 2013. Effects of global warming on hypoxia in the Baltic Sea–North Sea transition zone. Ecological Modelling 264:17–26. doi:10.1016/j.ecolmodel.2012.06.018.

[32] Keeling RF, Körtzinger A, Gruber N. 2009. Ocean deoxygenation in a warming world. Annu Rev Mar Sci 2:199–229. doi:10.1146/annurev.marine.010908.163855. 21141663

[33] Chai C, Yu Z, Song X, Cao X. 2006. The status and characteristics of eutrophication in the Yangtze River (Changjiang) Estuary and the adjacent East China Sea, China. Hydrobiologia 563:313–328. doi:10.1007/s10750-006-0021-7.

[34] Bouloubassi I, Fillaux J, Saliot A. 2001. Hydrocarbons in surface sediments from the Changjiang (Yangtze river) estuary, East China Sea. Mar Pollut Bull 42:1335–1346. doi:10.1016/s0025-326x(01)00149-7. 11827121

[35] Che Y, He Q, Lin W-Q. 2003. The distributions of particulate heavy metals and its indication to the transfer of sediments in the Changjiang Estuary and Hangzhou Bay, China. Marine Pollution Bull 46:123–131. doi:10.1016/S0025-326X(02)00355-7. 12535978

[36] Wei H, He Y, Li Q, Liu Z, Wang H. 2007. Summer hypoxia adjacent to the Changjiang Estuary. J Marine Systems 67:292–303. doi:10.1016/j.jmarsys.2006.04.014.

[37] Wang H, Dai M, Liu J, Kao S-J, Zhang C, Cai W-J, Wang G, Qian W, Zhao M, Sun Z. 2016. Eutrophication-driven hypoxia in the East China Sea off the Changjiang Estuary. Environ Sci Technol 50:2255–2263. doi:10.1021/acs.est.5b06211. 26824328

[38] Li H, Chen J, Lu Y, Jin H, Wang K, Zhang H. 2011. Seasonal variation of DO and formation mechanism of bottom water hypoxia of Changjiang River Estuary. J Mar Sci 29:78–87.

[39] Liu J, Zhu S, Liu X, Yao P, Ge T, Zhang X-H. 2020. Spatiotemporal dynamics of the archaeal community in coastal sediments: assembly process and co-occurrence relationship. ISME J 14:1463–1416. doi:10.1038/s41396-020-0621-7. 32132664 PMC7242467

[40] Chen Y, Li S, Yu Z, Chen Y, Mi T, Zhen Y. 2020. Characteristics of the Bathyarchaeota community in surface sediments from the southern Yellow Sea and northern East China sea. Estuarine, Coastal and Shelf Science 235:106595. doi:10.1016/j.ecss.2020.106595.

[41] Dang H, Zhang X, Sun J, Li T, Zhang Z, Yang G. 2008. Diversity and spatial distribution of sediment ammonia-oxidizing crenarchaeota in response to estuarine and environmental gradients in the Changjiang Estuary and East China Sea. Microbiology (Reading) 154:2084–2095. doi:10.1099/mic.0.2007/013581-0. 18599836

[42] Liu J, Liu X, Wang M, Qiao Y, Zheng Y, Zhang X-H. 2015. Bacterial and archaeal communities in sediments of the north Chinese marginal seas. Microb Ecol 70:105–117. doi:10.1007/s00248-014-0553-8. 25501892

[43] Edwards KJ, Gihring TM, Banfield JF. 1999. Seasonal Variations in Microbial Populations and Environmental Conditions in an Extreme Acid Mine Drainage Environment. Appl Environ Microbiol 65:3627–3632. doi:10.1128/AEM.65.8.3627-3632.1999. 10427059 PMC91544

[44] Li M, He H, Mi T, Zhen Y. 2022. Spatiotemporal dynamics of ammonia-oxidizing archaea and bacteria contributing to nitrification in sediments from Bohai Sea and South Yellow Sea, China. Sci Total Environ 825:153972. doi:10.1016/j.scitotenv.2022.153972. 35189237

[45] Yadav AN, Gulati S, Sharma D, Singh RN, Rajawat MVS, Kumar R, Dey R, Pal KK, Kaushik R, Saxena AK. 2019. Seasonal variations in culturable archaea and their plant growth promoting attributes to predict their role in establishment of vegetation in Rann of Kutch. Biologia 74:1031–1043. doi:10.2478/s11756-019-00259-2.

[46] Könneke M, Schubert DM, Brown PC, Hügler M, Standfest S, Schwander T, von Borzyskowski LS, Erb TJ, Stahl DA, Berg IA. 2014. Ammonia-oxidizing archaea use the most energy-efficient aerobic pathway for CO2 fixation. Proc Natl Acad Sci USA 111:8239–8244. doi:10.1073/pnas.1402028111. 24843170 PMC4050595

[47] Alves RJE, Minh BQ, Urich T, von Haeseler A, Schleper C. 2018. Unifying the global phylogeny and environmental distribution of ammonia-oxidising archaea based on amoA genes. Nat Commun 9:1–17. doi:10.1038/s41467-018-03861-1. 29666365 PMC5904100

[48] Cheung S, Mak W, Xia X, Lu Y, Cheung Y, Liu H. 2019. Overlooked genetic diversity of ammonia oxidizing archaea lineages in the global oceans. J Geophys Res Biogeosci 124:1799–1811. doi:10.1029/2018JG004636.

[49] Stahl DA, de la Torre JR. 2012. Physiology and diversity of ammonia-oxidizing archaea. Annu Rev Microbiol 66:83–101. doi:10.1146/annurev-micro-092611-150128. 22994489

[50] Qin W, Zheng Y, Zhao F, Wang Y, Urakawa H, Martens-Habbena W, Liu H, Huang X, Zhang X, Nakagawa T, Mende DR, Bollmann A, Wang B, Zhang Y, Amin SA, Nielsen JL, Mori K, Takahashi R, Virginia Armbrust E, Winkler M-KH, DeLong EF, Li M, Lee P-H, Zhou J, Zhang C, Zhang T, Stahl DA, Ingalls AE. 2020. Alternative strategies of nutrient acquisition and energy conservation map to the biogeography of marine ammonia-oxidizing archaea. ISME J: 14:2595–2515. doi:10.1038/s41396-020-0710-7. 32636492 PMC7490402

[51] Erguder TH, Boon N, Wittebolle L, Marzorati M, Verstraete W. 2009. Environmental factors shaping the ecological niches of ammonia-oxidizing archaea. FEMS Microbiol Rev 33:855–869. doi:10.1111/j.1574-6976.2009.00179.x. 19453522

[52] Inagaki F, Suzuki M, Takai K, Oida H, Sakamoto T, Aoki K, Nealson KH, Horikoshi K. 2003. Microbial communities associated with geological horizons in coastal subseafloor sediments from the Sea of Okhotsk. Appl Environ Microbiol 69:7224–7235. doi:10.1128/AEM.69.12.7224-7235.2003. 14660370 PMC309994

[53] Feng X, Wang Y, Zubin R, Wang F. 2019. Core metabolic features and hot origin of Bathyarchaeota. Engineering 5:498–504. doi:10.1016/j.eng.2019.01.011.

[54] Levin L, Ekau W, Gooday A, Jorissen F, Middelburg J, Naqvi S, Neira C, Rabalais N, Zhang J. 2009. Effects of natural and human-induced hypoxia on coastal benthos. Biogeosciences 6:2063–2098. doi:10.5194/bg-6-2063-2009.

[55] Zhang Y, Xie X, Jiao N, Hsiao S-Y, Kao S-J. 2014. Diversity and distribution of amoA-type nitrifying and nirS-type denitrifying microbial communities in the Yangtze River estuary. Biogeosciences 11:2131–2145. doi:10.5194/bg-11-2131-2014.

[56] Zheng Y, Hou L, Newell S, Liu M, Zhou J, Zhao H, You L, Cheng X. 2014. Community dynamics and activity of ammonia-oxidizing prokaryotes in intertidal sediments of the Yangtze Estuary. Appl Environ Microbiol 80:408–419. doi:10.1128/AEM.03035-13. 24185847 PMC3911015

[57] Ren M, Feng X, Huang Y, Wang H, Hu Z, Clingenpeel S, Swan BK, Fonseca MM, Posada D, Stepanauskas R, Hollibaugh JT, Foster PG, Woyke T, Luo H. 2019. Phylogenomics suggests oxygen availability as a driving force in Thaumarchaeota evolution. ISME J 13:2150–2161. doi:10.1038/s41396-019-0418-8. 31024152 PMC6776046

[58] He H, Zhen Y, Mi T, Yu Z. 2016. Community composition and abundance of ammonia-oxidizing archaea in sediments from the Changjiang estuary and its adjacent area in the East China Sea. Geomicrobiol J 33:416–425. doi:10.1080/01490451.2014.986695.

[59] Zou D, Li Y, Kao SJ, Liu H, Li M. 2019. Genomic adaptation to eutrophication of ammonia-oxidizing archaea in the Pearl River estuary. Environ Microbiol 21:2320–2332. doi:10.1111/1462-2920.14613. 30924222

[60] Zou D, Wan R, Han L, Xu MN, Liu Y, Liu H, Kao S-J, Li M. 2020. Genomic characteristics of a novel species of ammonia-oxidizing Archaea from the Jiulong River Estuary. Appl Environ Microbiol 86:e00736-20. doi:10.1128/AEM.00736-20. 32631866 PMC7480389

[61] Zou D, Pan J, Liu Z, Zhang C, Liu H, Li M. 2020. The distribution of Bathyarchaeota in surface sediments of the Pearl river estuary along salinity gradient. Front Microbiol 11:285. doi:10.3389/fmicb.2020.00285. 32174899 PMC7056671

[62] Pan J, Chen Y, Wang Y, Zhou Z, Li M. 2019. Vertical distribution of Bathyarchaeotal communities in mangrove wetlands suggests distinct niche preference of Bathyarchaeota subgroup 6. Microb Ecol 77:417–428. doi:10.1007/s00248-018-1309-7. 30612184

[63] Fillol M, Sànchez-Melsió A, Gich F, Borrego C. 2015. Diversity of Miscellaneous Crenarchaeotic Group archaea in freshwater karstic lakes and their segregation between planktonic and sediment habitats. FEMS Microbiol Ecol 91:fiv020. doi:10.1093/femsec/fiv020. 25764468

[64] Martens-Habbena W, Berube P, Urakawa H, de La Torre J, Stahl D. 2009. Ammonia oxidation kinetics determine niche separation of nitrifying Archaea and Bacteria. Nature 461:976–979. doi:10.1038/nature08465. 19794413

[65] Lazar CS, Baker BJ, Seitz K, Hyde AS, Dick GJ, Hinrichs KU, Teske AP. 2016. Genomic evidence for distinct carbon substrate preferences and ecological niches of B athyarchaeota in estuarine sediments. Environ Microbiol 18:1200–1211. doi:10.1111/1462-2920.13142. 26626228

[66] Zhou Z, Liu Y, Lloyd KG, Pan J, Yang Y, Gu J-D, Li M. 2019. Genomic and transcriptomic insights into the ecology and metabolism of benthic archaeal cosmopolitan, Thermoprofundales (MBG-D archaea). ISME J 13:885–901. doi:10.1038/s41396-018-0321-8. 30514872 PMC6461988

[67] Zhou Z, Liu Y, Xu W, Pan J, Luo Z-H, Li M. 2020. Genome-and community-level interaction insights into carbon utilization and element cycling functions of Hydrothermarchaeota in hydrothermal sediment. Msystems 5. doi:10.1128/mSystems.00795-19. PMC6946796 31911466

[68] Yu T, Liang Q, Niu M, Wang F. 2017. High occurrence of Bathyarchaeota (MCG) in the deep-sea sediments of South China Sea quantified using newly designed PCR primers. Environ Microbiol Rep 9:374–382. doi:10.1111/1758-2229.12539. 28419783

[69] Seyler LM, McGuinness LM, Kerkhof LJ. 2014. Crenarchaeal heterotrophy in salt marsh sediments. ISME J 8:1534–1543. doi:10.1038/ismej.2014.15. 24553469 PMC4069397

[70] Fu L, Chen Y, Li S, He H, Mi T, Zhen Y, Yu Z. 2019. Shifts in the anammox bacterial community structure and abundance in sediments from the Changjiang Estuary and its adjacent area. Systematic and Applied Microbiology 42:383–396. doi:10.1016/j.syapm.2018.12.008. 30679000

[71] Callahan BJ, McMurdie PJ, Rosen MJ, Han AW, Johnson AJA, Holmes SP. 2016. DADA2: high-resolution sample inference from Illumina amplicon data. Nat Methods 13:581–583. doi:10.1038/nmeth.3869. 27214047 PMC4927377

[72] Bolyen E, Rideout JR, Dillon MR, Bokulich NA, Abnet CC, Al-Ghalith GA, Alexander H, Alm EJ, Arumugam M, Asnicar F, Bai Y, Bisanz JE, Bittinger K, Brejnrod A, Brislawn CJ, Brown CT, Callahan BJ, Caraballo-Rodríguez AM, Chase J, Cope EK, Da Silva R, Diener C, Dorrestein PC, Douglas GM, Durall DM, Duvallet C, Edwardson CF, Ernst M, Estaki M, Fouquier J, Gauglitz JM, Gibbons SM, Gibson DL, Gonzalez A, Gorlick K, Guo J, Hillmann B, Holmes S, Holste H, Huttenhower C, Huttley GA, Janssen S, Jarmusch AK, Jiang L, Kaehler BD, Kang KB, Keefe CR, Keim P, Kelley ST, Knights D, et al. 2019. Reproducible, interactive, scalable and extensible microbiome data science using QIIME 2. Nat Biotechnol 37:852–857. doi:10.1038/s41587-019-0209-9. 31341288 PMC7015180

[73] Quast C, Pruesse E, Yilmaz P, Gerken J, Schweer T, Yarza P, Peplies J, Glöckner FO. 2013. The SILVA ribosomal RNA gene database project: improved data processing and web-based tools. Nucleic Acids Res 41:D590–D596. doi:10.1093/nar/gks1219. 23193283 PMC3531112

[74] Wemheuer F, Taylor JA, Daniel R, Johnston E, Meinicke P, Thomas T, Wemheuer B. 2020. Tax4Fun2: prediction of habitat-specific functional profiles and functional redundancy based on 16S rRNA gene sequences. Environ Microbiome 15:11–12. doi:10.1186/s40793-020-00358-7. 33902725 PMC8067651

[75] Caporaso JG, Lauber CL, Walters WA, Berg-Lyons D, Lozupone CA, Turnbaugh PJ, Fierer N, Knight R. 2011. Global patterns of 16S rRNA diversity at a depth of millions of sequences per sample. Proc Natl Acad Sci USA 108:4516–4522. doi:10.1073/pnas.1000080107. 20534432 PMC3063599

[76] Ovreås L, Forney L, Daae FL, Torsvik V. 1997. Distribution of bacterioplankton in meromictic Lake Saelenvannet, as determined by denaturing gradient gel electrophoresis of PCR-amplified gene fragments coding for 16S rRNA. Appl Environ Microbiol 63:3367–3373. doi:10.1128/aem.63.9.3367-3373.1997. 9292986 PMC168642

[77] Jorgensen SL, Hannisdal B, Lanzén A, Baumberger T, Flesland K, Fonseca R, Ovreås L, Steen IH, Thorseth IH, Pedersen RB, Schleper C. 2012. Correlating microbial community profiles with geochemical data in highly stratified sediments from the Arctic Mid-Ocean Ridge. Proc Natl Acad Sci USA 109:E2846–E2855. doi:10.1073/pnas.1207574109. 23027979 PMC3479504

[78] Hong J-K, Kim H-J, Cho J-C. 2014. Novel PCR primers for the archaeal phylum Thaumarchaeota designed based on the comparative analysis of 16S rRNA gene sequences. PLoS One 9:e96197. doi:10.1371/journal.pone.0096197. 24805255 PMC4013054

[79] Zhou Z, Zhang G-X, Xu Y-B, Gu J-D. 2018. Successive transitory distribution of Thaumarchaeota and partitioned distribution of Bathyarchaeota from the Pearl River estuary to the northern South China Sea. Appl Microbiol Biotechnol 102:8035–8048. doi:10.1007/s00253-018-9147-6. 29946932

[80] Zhu Z-Y, Zhang J, Wu Y, Zhang Y-Y, Lin J, Liu S-M. 2011. Hypoxia off the Changjiang (Yangtze River) estuary: oxygen depletion and organic matter decomposition. Marine Chemistry 125:108–116. doi:10.1016/j.marchem.2011.03.005.

[81] Nguyen L-T, Schmidt HA, Von Haeseler A, Minh BQ. 2015. IQ-TREE: a fast and effective stochastic algorithm for estimating maximum-likelihood phylogenies. Mol Biol Evol 32:268–274. doi:10.1093/molbev/msu300. 25371430 PMC4271533

[82] Letunic I, Bork P. 2019. Interactive Tree Of Life (iTOL) v4: recent updates and new developments. Nucleic Acids Res 47:W256–W259. doi:10.1093/nar/gkz239. 30931475 PMC6602468

