# Supplementary material for: Distinct Features of Sedimentary Archaeal Communities in Hypoxia and Non-Hypoxia Regions off the Changjiang River Estuary
Source: Microbiol Spectr. 2022 Sep 6;10(5):e01947-22. doi: 10.1128/spectrum.01947-22 (PMC9602602; doi:10.1128/spectrum.01947-22)
Supplement: Supplemental file 1 — Tables S1 to S4 and Fig. S1 to S11. Download spectrum.01947-22-s0001.pdf, PDF file, 2.4 MB [file spectrum.01947-22-s0001.pdf]

Supplementary information

Distinct features of sedimentary archaeal communities in hypoxia and non-hypoxia regions off the Changjiang River Estuary

Dayu Zou[1,2,5], Hongliang Li[3,6,7], Ping Du[3], Bing Wang[3], Hua Lin[3], Hongbin Liu[4], Jianfang Chen[3], Meng Li[1,5*]

[1]Archaeal Biology Center, Institute for Advanced Study, Shenzhen University, Shenzhen 518060, China

[2]Key Laboratory of Optoelectronic Devices and Systems, College of Physics and Optoelectronic Engineering, Shenzhen University, Shenzhen 518060, China

[3]Key Laboratory of Marine Ecosystem Dynamics, Second Institute of Oceanography, Ministry of Natural Resources, Hangzhou 310012, China

[4]Department of Ocean Science and Hong Kong Branch of Southern Marine Science & Engineering Guangdong Laboratory (Guangzhou), The Hong Kong University of Science and Technology, Hong Kong SAR, China

[5]Shenzhen Key Laboratory of Marine Microbiome Engineering, Institute for Advanced Study, Shenzhen University, Shenzhen, Guangdong, China

[6]Key Laboratory of Marine Ecosystem Dynamics, Second Institute of Oceanography, Ministry of Natural Resources, Hangzhou, China

[7]Southern Marine Science and Engineering Guangdong Laboratory (Zhuhai), Zhuhai, China

*** Correspondence:** Meng Li (limeng848@szu.edu.cn)

Running Title: The archaeal community in hypoxia estuarine sediments.

Keywords: Thaumarchaeota, Bathyarchaeota, hypoxia, distribution

Tables

Table S1 Station information and environmental factors used in this study.

Station	Longitude [°E]	Latitude [°N]	Bottom Depth	TOC (mg/g)	TN (mg/g)	Sulfide (μg/g)	Salinity	DO saturation (%)	pH	NH4+ (μ mol/L)	NO3- (μ mol/L)	NO2- (μ mol/L)	PO4- (μ mol/L)
N1	122.010	32.435	22	4.3	0.55	2.76	30.98	1.13	7.73	10.49	BDL	BDL	0.46
N3	122.659	32.550	32	3.5	0.44	1.71	31.73	5.65	7.86	6.73	2.63	3.87	0.57
N5	123.315	32.702	35	2.3	0.24	4.95	30.64	18.69	7.75	3.54	12.63	2.35	0.42
N7	124.003	32.819	43	0.7	0.05	5.11	31.24	39.17	7.89	3.08	16.09	0.18	0.53
J1	122.139	32.006	15	8.0	0.95	3.82	27.87	24.15	7.76	4.13	6.14	3.08	0.12
J3	122.491	32.025	24	6.1	0.73	5.53	31.97	5.72	7.77	8.00	0.30	0.03	0.58
J5	123.197	32.104	37	4.9	0.45	4.03	32.96	18.90	7.76	1.18	10.40	2.47	0.63
J7	124.152	32.214	40	2.6	0.16	2.92	31.02	47.65	8.21	2.44	12.61	0.18	0.26
A1	122.237	31.503	10	4.1	0.58	0.64	27.45	20.16	7.65	7.68	6.65	1.18	0.74
A3	122.606	31.500	34	3.6	0.4	1.25	34.11	36.08	7.83	0.63	-	23.52	0.85
A5	123.001	31.496	33	1.5	0.14	4.01	33.79	44.21	7.9	1.10	12.39	0.76	0.65
A7	123.408	31.501	36	1.2	0.15	4.32	33.30	56.29	7.95	0.92	10.58	0.22	0.52
B2	121.869	31.152	8	4.4	-	-	2.72	76.30	7.73	3.20	108.90	0.63	1.51
B5	122.400	31.005	9	5.2	0.63	5.09	19.66	44.41	7.76	2.60	49.71	0.74	0.74
B8	122.999	30.992	48	6.0	0.79	4.74	34.46	37.90	7.83	1.46	14.18	0.60	0.84
B10	123.396	31.000	54	2.1	0.32	4.38	34.50	38.63	7.84	1.39	12.03	0.31	0.85
B11	123.745	31.001	47	2.2	0.22	4.67	34.52	34.80	7.85	0.89	9.69	0.28	0.85
C1	122.199	30.495	12	4.8	0.63	2.44	23.42	71.98	7.93	2.16	45.98	0.15	1.04
C3	122.667	30.501	33	3.2	0.94	5.91	19.70	18.52	7.81	7.98	27.43	1.10	0.86
C5	123.016	30.498	61	2.1	0.39	2.21	30.70	24.45	7.86	6.48	22.50	0.29	1.09
C7	123.404	30.499	58	2.0	0.49	1.73	33.41	49.15	7.84	1.54	13.23	0.11	0.90
D1	122.501	30.008	20	2.6	0.87	3.65	27.95	54.22	7.95	4.48	13.49	1.67	0.56
D3	122.806	30.003	42	2.4	0.79	2.38	34.25	43.59	7.79	5.12	20.04	0.67	0.95
D5	123.191	30.000	60	2.7	0.21	3.13	34.79	28.18	7.81	5.41	20.20	0.26	0.95
E1	122.304	29.586	15	4.2	0.93	2.96	27.98	74.63	7.99	1.70	27.52	3.08	0.74
E3	122.544	29.521	35	3.6	0.74	3.27	33.36	19.05	7.82	2.35	15.86	1.80	1.02
E5	122.906	29.469	59	4.8	0.65	2.67	34.82	48.64	7.79	4.72	21.74	0.17	1.05
F1	122.104	29.065	12	5.6	0.74	3.27	27.95	104.63	8.28	2.19	10.94	1.73	0.08

F3	122.342	28.990	34	5.5	0.65	2.67	33.66	21.34	7.71	3.07	20.59	0.50	1.26
F5	122.690	28.916	60	2.5	0.66	2.08	34.55	61.50	7.91	2.45	13.88	0.05	0.87

*Environmental factors were retrieved from previous studies (Lin et al., 2016; Zhao et al., 2019; Wang and Gao, 2022).

**BDL, below detection limit; -, not measured.

Table S2 Detailed information for qPCR experiments.

Target gene	Target description	Primer name	Sequence (5'-3')	Annealing temperature	R2	Efficiency (%)
16S rRNA	Bacteria+Archaea	Uni515F	GTGYCAGCMGCCGCGGTAA	60° C	0.998	89.97
		Uni806R	GGACTACNVGGGTWTCTAAT			
16S rRNA	Archaea	Arch519F	CAGCCGCCGCGGTAA	60° C	0.998	89.71
		Arch908R	CCCGCCAATTCCTTTAAGTT			
16S rRNA	*Thaumarchaeota*	Thaum494	GAATAAGGGGTGGGCAAGT	56° C	0.995	87.7
		Arch958R	YCCGGCGTTGAVTCCAATT			
16S rRNA	*Bathyarchaeota*	MCG242dF	TDACCGGTDCGGGCCGTG	60° C	0.999	94.8
		Bathy442R	GGCGGCTGACACCAGTCT			

Table S3 Sequencing information, the diversity index, and qPCR results of samples.

Sample	Clean archaeal reads	Shannon index	Simpson index	OTUs	Thaumarchaeotal OTUs	Bathyarchaeota OTUs	16S rRNA gene abundance of	16S rRNA gene abundance of total archaea*	16S rRNA gene abundance of	16S rRNA gene abundance of

							total prokaryotes*		Thaumarchaeota*	Bathyarchaeota*
A1	58,647	6.2231	0.9588	384	109	163	9.2218(0.0280)	8.6247(0.0181)	8.4846(0.1087)	7.2967(0.0058)
A3	51,251	5.1535	0.9345	241	89	73	9.2252(0.0095)	8.7700(0.0411)	8.7042(0.0889)	6.3298(0.0080)
A5	50,667	5.5723	0.9422	294	94	117	9.7253(0.0765)	9.0432(0.0424)	8.4362(0.0767)	6.4939(0.0117)
A7	54,051	6.4587	0.9666	415	91	220	8.8620(0.0084)	8.6243(0.0077)	8.4260(0.0479)	5.6905(0.0544)
B2	50,264	6.7816	0.9716	445	86	210	8.3882(0.0230)	7.9006(0.0497)	6.9320(0.0247)	6.6056(0.0189)
B5	51,860	6.2796	0.9558	425	96	230	9.0579(0.0143)	8.6186(0.0075)	8.2604(0.0020)	7.4292(0.0323)
B8	52,453	6.2131	0.9543	404	91	203	8.9861(0.0317)	8.5213(0.0377)	8.3450(0.0175)	7.3810(0.0214)
B10	58,096	5.9889	0.9540	376	95	150	8.6368(0.0267)	8.1427(0.0094)	7.9055(0.0394)	6.2882(0.0652)
B11	49,767	5.3921	0.9341	280	96	119	9.0819(0.0092)	8.7866(0.0099)	8.6655(0.0545)	6.7213(0.0168)
C1	50,727	5.3045	0.9303	268	87	123	9.3889(0.0280)	9.0281(0.0110)	8.8155(0.0362)	7.7738(0.0214)
C3	54,633	7.0069	0.9773	492	77	298	9.4590(0.0274)	9.2581(0.0324)	7.9527(0.0257)	8.5437(0.0138)
C5	54,660	7.0600	0.9715	555	76	302	9.4602(0.0181)	8.8121(0.0210)	7.7075(0.0935)	7.5274(0.0046)
C7	55,923	6.2678	0.9559	438	99	254	9.3867(0.0095)	8.9291(0.0531)	8.5518(0.0723)	7.1448(0.0435)
D1	57,787	6.0040	0.9418	412	86	226	9.3601(0.0447)	8.9965(0.0189)	8.6691(0.0154)	8.0451(0.0090)
D3	53,348	5.7681	0.9391	376	80	188	9.1818(0.0109)	8.8780(0.0128)	8.4272(0.0734)	7.5984(0.0160)
D5	55,133	5.6106	0.9338	364	82	169	8.9907(0.0263)	8.7795(0.0093)	8.5484(0.0473)	7.2850(0.0324)
E1	52,820	6.0213	0.9511	378	82	162	9.2301(0.0259)	9.0219(0.0352)	8.4467(0.0420)	7.7350(0.0381)
E3	40,982	5.8400	0.9509	287	59	92	9.2041(0.0086)	8.8833(0.0171)	8.1479(0.0183)	7.7300(0.0139)
E5	42,422	5.7427	0.9412	324	57	133	9.1001(0.0477)	8.9102(0.0124)	8.3197(0.0266)	7.6900(0.0062)
F1	44,125	5.2950	0.9193	278	54	96	9.4139(0.0203)	9.1946(0.0102)	8.6809(0.0474)	7.6152(0.0032)
F3	50,711	5.8803	0.9395	353	58	119	9.3302(0.0180)	8.9772(0.0151)	8.1669(0.0309)	7.4107(0.0253)
F5	46,289	5.2541	0.9275	273	55	98	9.2307(0.0410)	9.0449(0.0103)	8.6786(0.0325)	7.5375(0.0082)
J1	46,611	5.9157	0.9450	313	50	129	9.2997(0.0440)	8.7842(0.0328)	8.5434(0.0005)	7.7240(0.0370)
J3	43,013	5.8456	0.9518	275	44	101	9.2576(0.0341)	8.7392(0.0061)	8.6313(0.0135)	7.3191(0.0495)
J5	32,702	4.9124	0.9323	145	64	29	8.5401(0.0231)	8.1769(0.0038)	8.0886(0.0346)	5.5019(0.0644)
J7	49,586	5.4302	0.9346	321	63	131	9.1049(0.0291)	8.5334(0.0211)	8.4078(0.0161)	6.7383(0.0162)
N1	45,559	6.1852	0.9243	374	32	123	9.4167(0.0292)	8.7100(0.0409)	7.8703(0.0039)	7.8388(0.0453)
N3	43,550	4.4769	0.8851	200	55	70	9.3981(0.0167)	8.9244(0.0191)	8.8758(0.0325)	6.7407(0.0509)
N5	44,259	7.1638	0.9793	442	44	258	9.0683(0.0353)	8.6548(0.0518)	8.2249(0.0421)	7.4805(0.0269)
N7	46,316	6.0740	0.9504	344	54	194	8.9755(0.0561)	8.4311(0.0185)	8.2179(0.0221)	7.0583(0.0236)

*The abundance of 16S rRNA gene was log10 transformed, with the standard deviation in the bracket. The unit is gene copies per gram dry sediments.

Table S4 Detailed archaeal community composition of samples.

Sample	Thaumarchaeota	Bathyarchaeota	Euryarchaeota	Lokiarchaeota	Hydrothermarch aeota	Odinarchaeota	Other
A1	0.6663	0.2450	0.0490	0.0134	0.0148	0.0023	0.0092
A3	0.8615	0.0780	0.0333	0.0120	0.0045	0.0035	0.0072
A5	0.7659	0.1248	0.0436	0.0388	0.0091	0.0107	0.0070
A7	0.6096	0.2807	0.0644	0.0220	0.0071	0.0046	0.0115
B2	0.5232	0.2422	0.1889	0.0168	0.0090	0.0037	0.0163
B5	0.6420	0.2390	0.0537	0.0219	0.0190	0.0041	0.0204
B8	0.6252	0.2358	0.0719	0.0215	0.0153	0.0090	0.0213
B10	0.7049	0.1940	0.0566	0.0141	0.0137	0.0041	0.0127
B11	0.7794	0.1552	0.0284	0.0164	0.0106	0.0035	0.0065
C1	0.7677	0.1421	0.0172	0.0136	0.0488	0.0028	0.0079
C3	0.3788	0.4867	0.0656	0.0266	0.0088	0.0050	0.0284
C5	0.4438	0.3472	0.1098	0.0287	0.0209	0.0122	0.0373
C7	0.6327	0.2402	0.0673	0.0228	0.0156	0.0045	0.0169
D1	0.6213	0.2750	0.0432	0.0223	0.0174	0.0041	0.0167
D3	0.6998	0.1856	0.0473	0.0218	0.0238	0.0060	0.0157
D5	0.7051	0.2001	0.0370	0.0193	0.0184	0.0044	0.0158
E1	0.6367	0.2293	0.0506	0.0191	0.0420	0.0054	0.0168
E3	0.6509	0.1840	0.0787	0.0317	0.0241	0.0090	0.0216
E5	0.6337	0.2151	0.0710	0.0288	0.0213	0.0075	0.0226
F1	0.7098	0.1473	0.0605	0.0201	0.0298	0.0074	0.0250
F3	0.6091	0.1583	0.0953	0.0243	0.0453	0.0287	0.0389
F5	0.7337	0.1257	0.0639	0.0235	0.0281	0.0059	0.0193
J1	0.5302	0.3183	0.0585	0.0310	0.0233	0.0077	0.0310
J3	0.5642	0.2812	0.0603	0.0252	0.0272	0.0121	0.0299
J5	0.8632	0.0671	0.0288	0.0076	0.0153	0.0015	0.0165
J7	0.7417	0.1465	0.0549	0.0217	0.0086	0.0098	0.0169
N1	0.1813	0.2653	0.4277	0.0763	0.0103	0.0095	0.0296
N3	0.8349	0.0931	0.0354	0.0149	0.0052	0.0053	0.0112
N5	0.3331	0.4611	0.0955	0.0575	0.0075	0.0117	0.0337

N7	0.5434	0.3068	0.0631	0.0426	0.0073	0.0079	0.0289

Figures

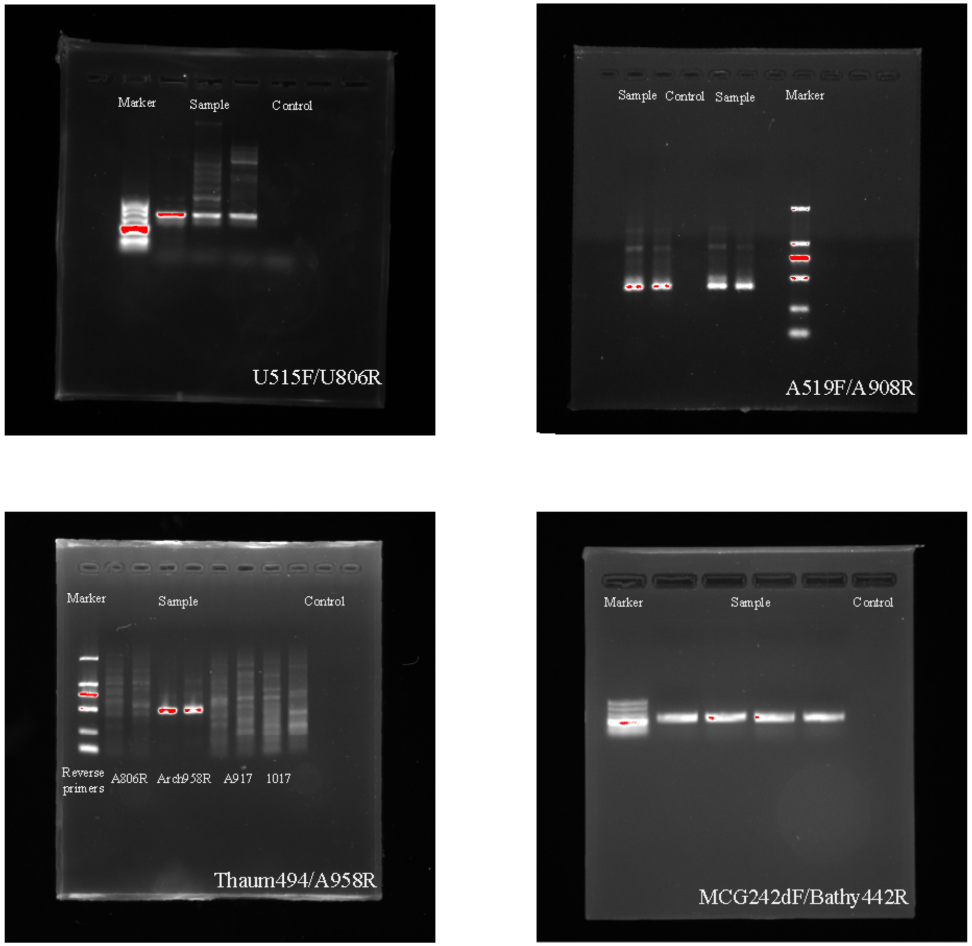

Figure S1 Gel-electrophoretograms of all primers used in the qPCR experiments in this study, including Uni515F/Uni806R, Arch519F/Arch908R, Thaum494/Arch958R, and MCG242dF/BATHY442R.

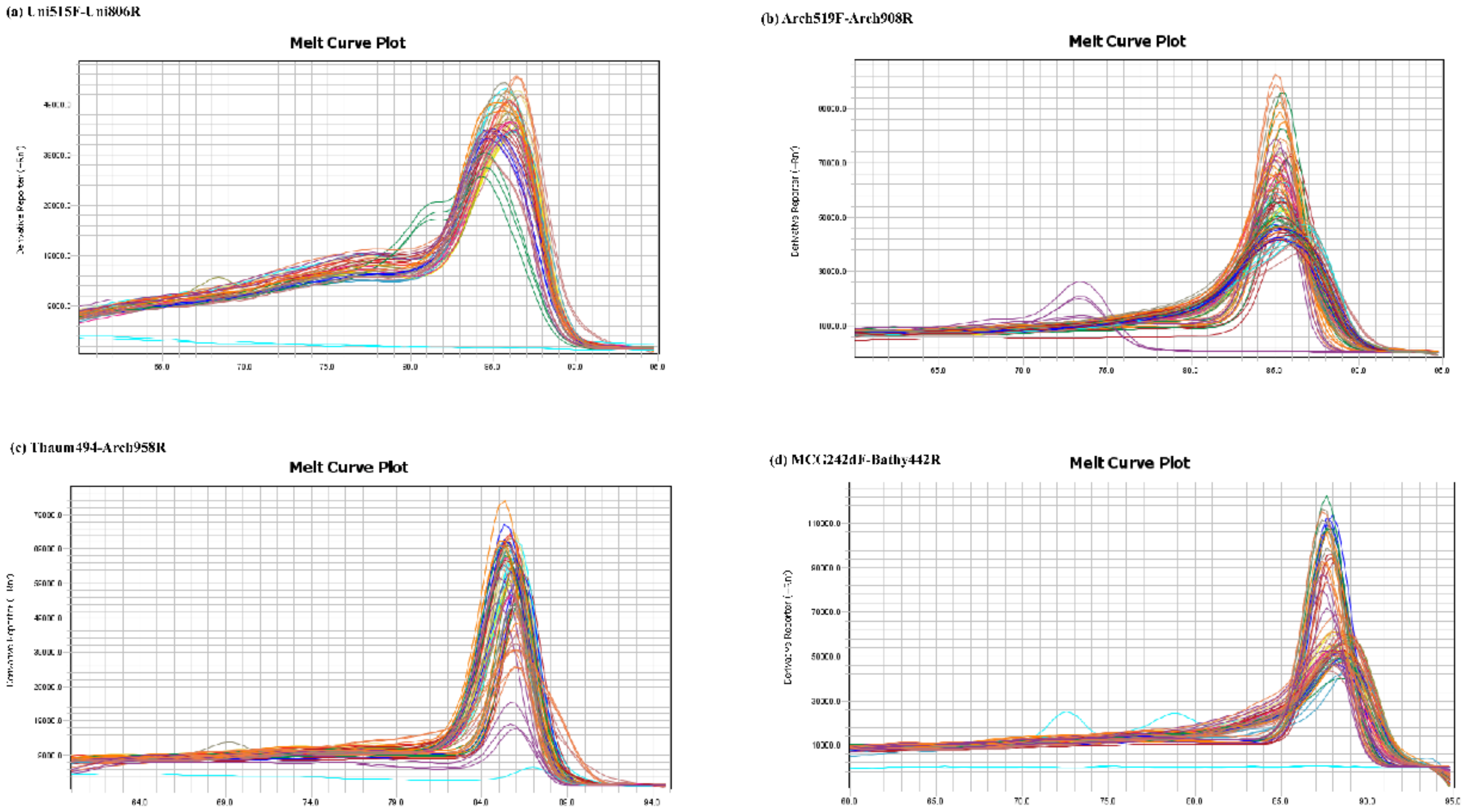

Figure S2 Melting curves of all qPCR experiments targeted at 16S rRNA genes of the total prokaryotes (a), the total archaea (b), *Thaumarchaeota* (c), and *Bathyarchaeota* (d).

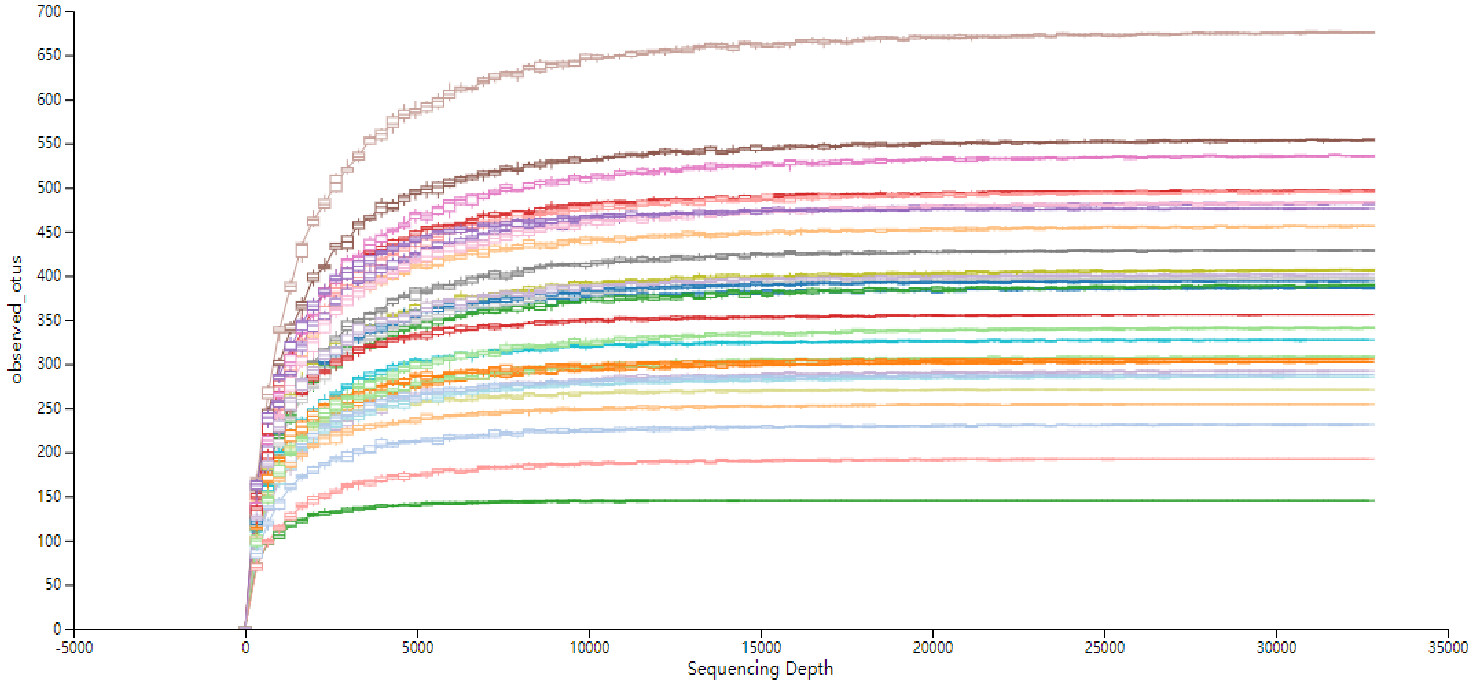

Figure S3 Rarefaction curves of the sequencing result in this study. All samples had reached the plateau at the normalization sequencing depth 32702.

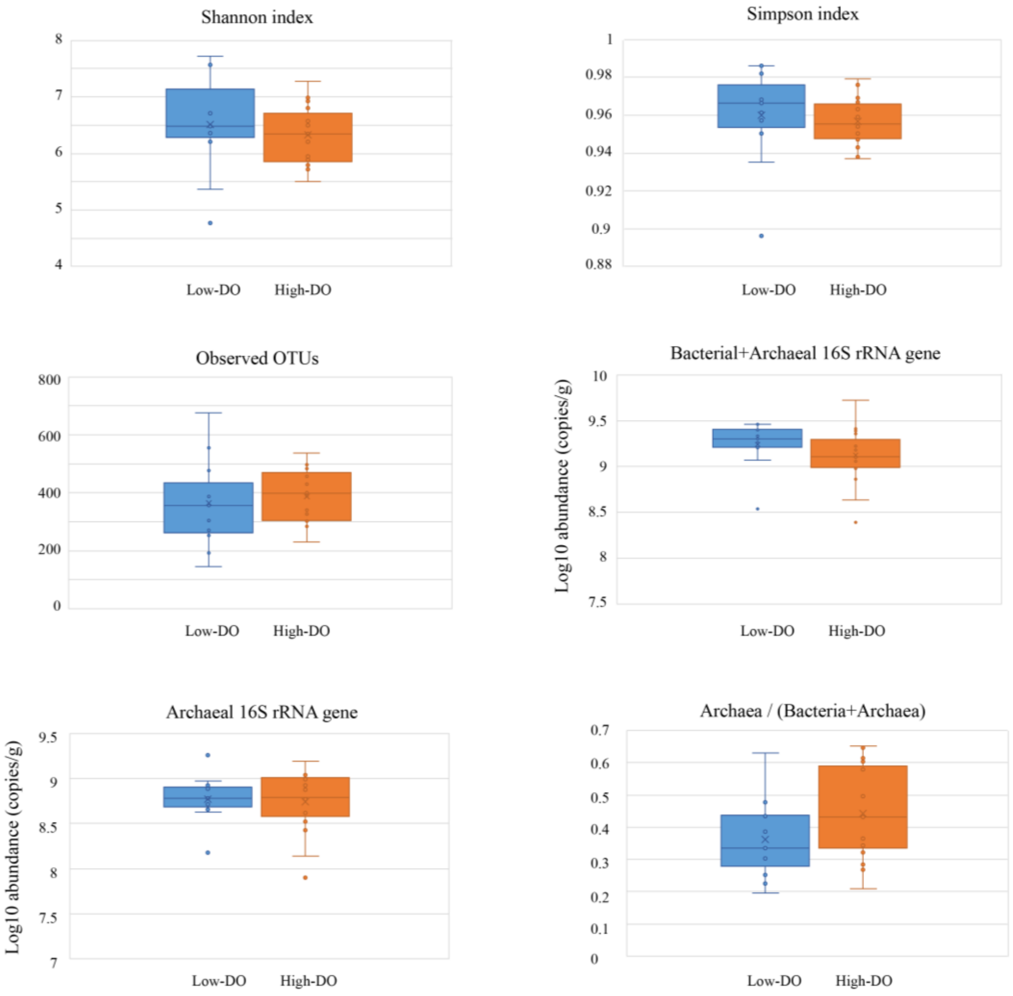
Shannon index
8
7
6
5
4
Low-DO
High-DO
Simpson index
1
0.98
0.96
0.94
0.92
0.9
0.88
Low-DO
High-DO
Observed OTUs
800
600
400
200
0
Low-DO
High-DO
Bacterial+Archaeal 16S rRNA gene
Log10 abundance (copies/g)
10
9.5
9
8.5
8
7.5
Low-DO
High-DO
Archaeal 16S rRNA gene
Log10 abundance (copies/g)
9.5
9
8.5
8
7.5
7
Low-DO
High-DO
Archaea / (Bacteria+Archaea)
0.7
0.6
0.5
0.4
0.3
0.2
0.1
0
Low-DO
High-DO

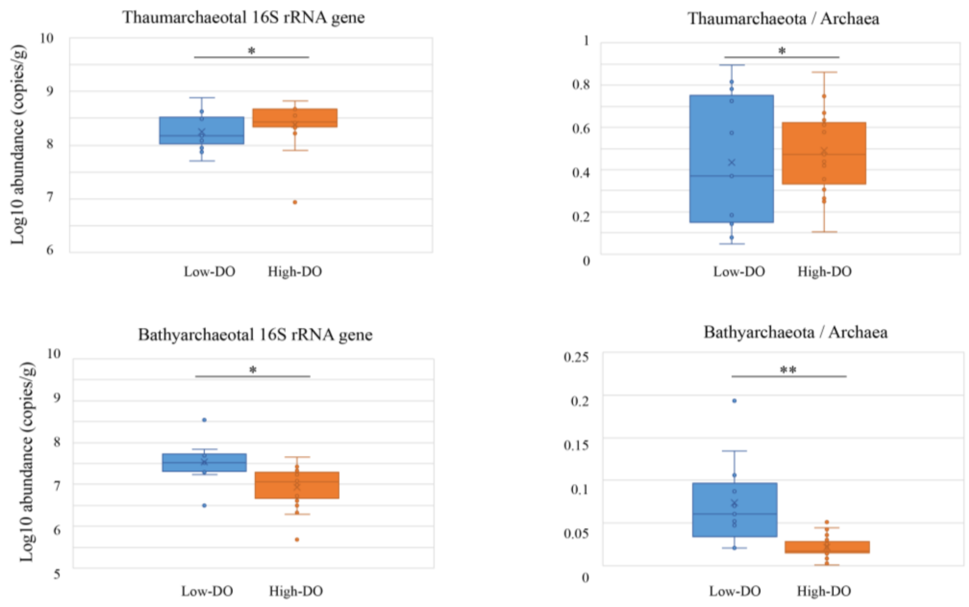

Figure S4 Comparison results of the community properties between the low- (blue) and high-DO (orange) samples, including the diversity index, the 16S rRNA gene abundance, and the abundance fraction.

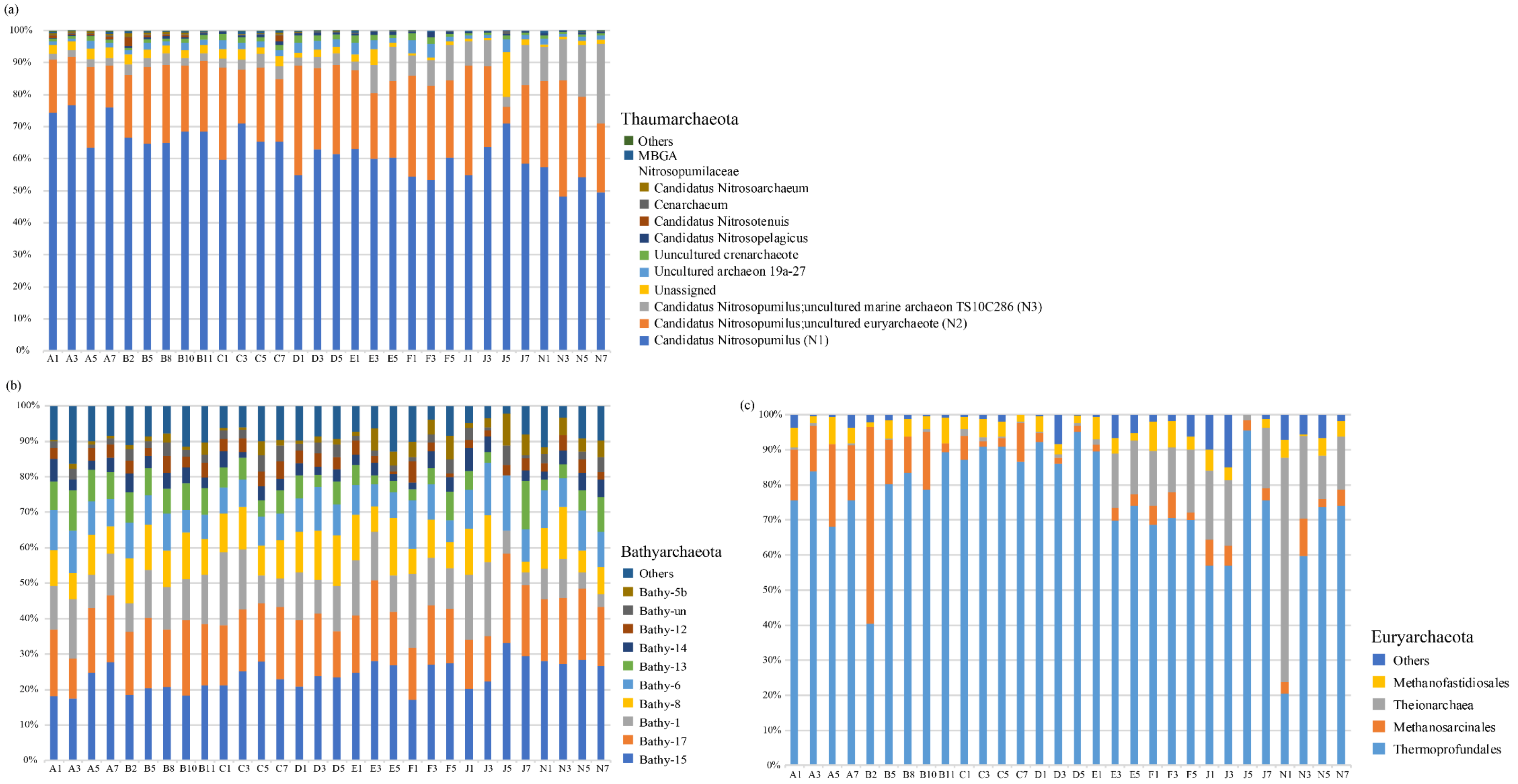

Figure S5 Detailed community composition of *Thaumarchaeota* (a), *Bathyarchaeota* (b), and *Euryarchaeota* (c) in this study.

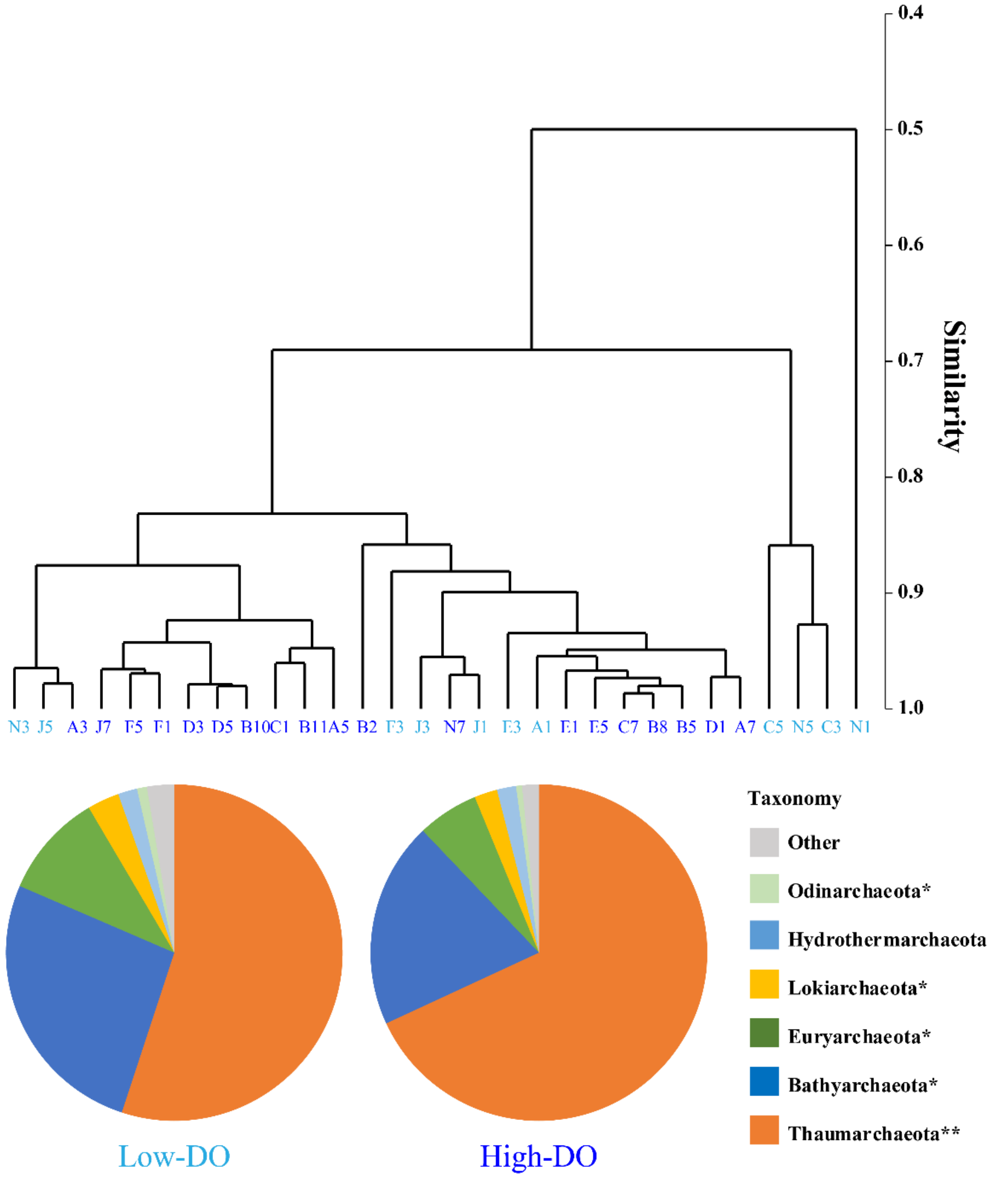

Figure S6 Sample clustering results (top) and the average archaeal community composition of the low- and high-DO group (bottom). The symbol * and ** indicate that there are significant differences ($p < 0.05$ and $p < 0.01$, respectively) between the low- and high-DO group.

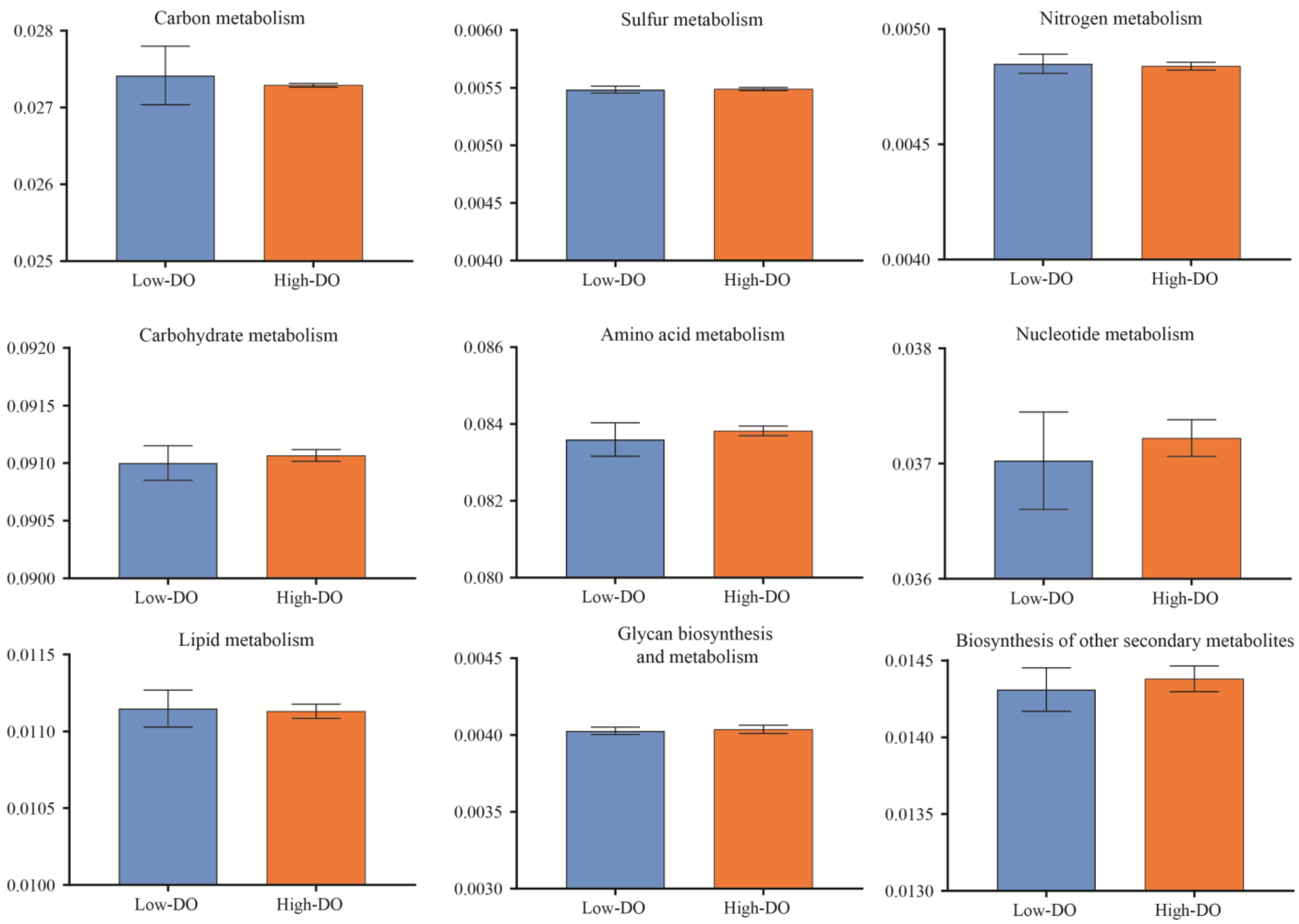

Figure S7 The comparison of Tax4Fun2 predicted community metabolic potentials between low- and high-DO samples. The y-axis represented the abundance fraction.

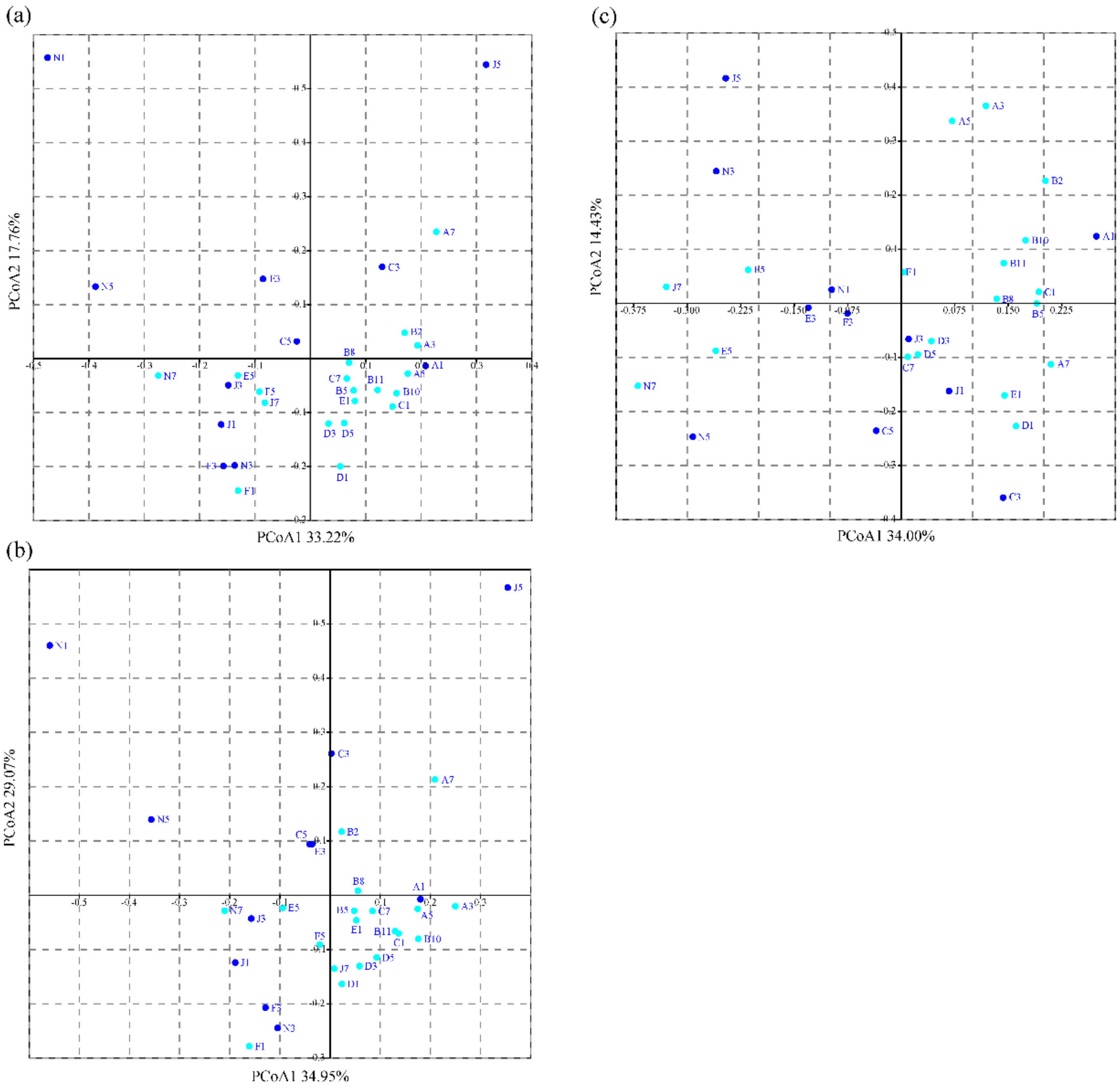

Figure S8 The PCoA plot about the distribution of samples based on community composition of the total archaeal (a), *Thaumarchaeota* (b), and *Bathyarchaeota* (c) in OTU level. Light blue dots stand for the high-DO group samples, and dark blue dots stand for the low-DO group.

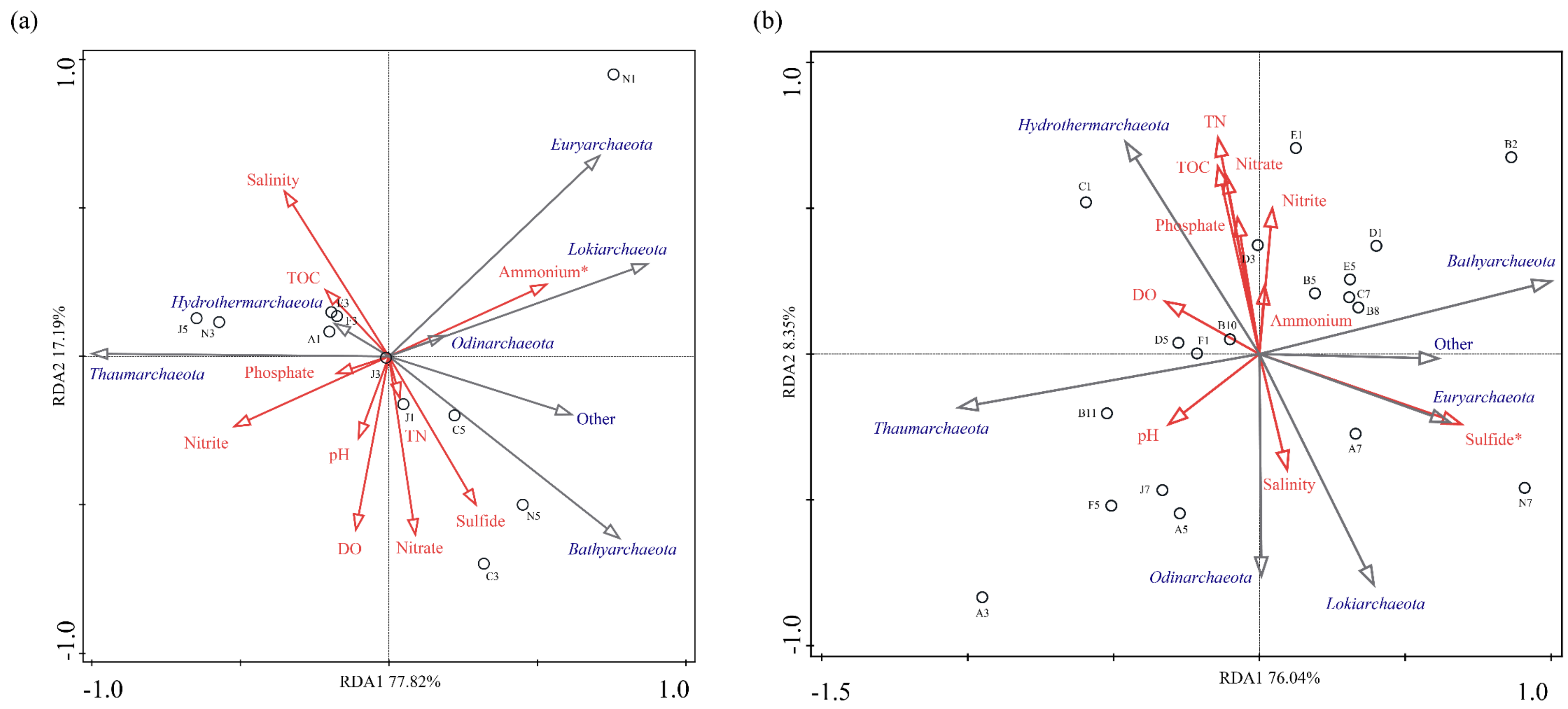

Figure S9 The db-RDA results of the archaeal community in low- (a) and high-DO (b) samples. Ammonium, nitrate, and salinity were the most important factor in the low-DO samples, explaining 23.7% ($p < 0.05$), 22.4%, and 16.0% of total variation, respectively. Sulfide, TN, and TOC were the most important factor in the high-DO samples, explaining 37.2% ($p < 0.05$), 9.4%, and 8.6% of total variation, respectively.

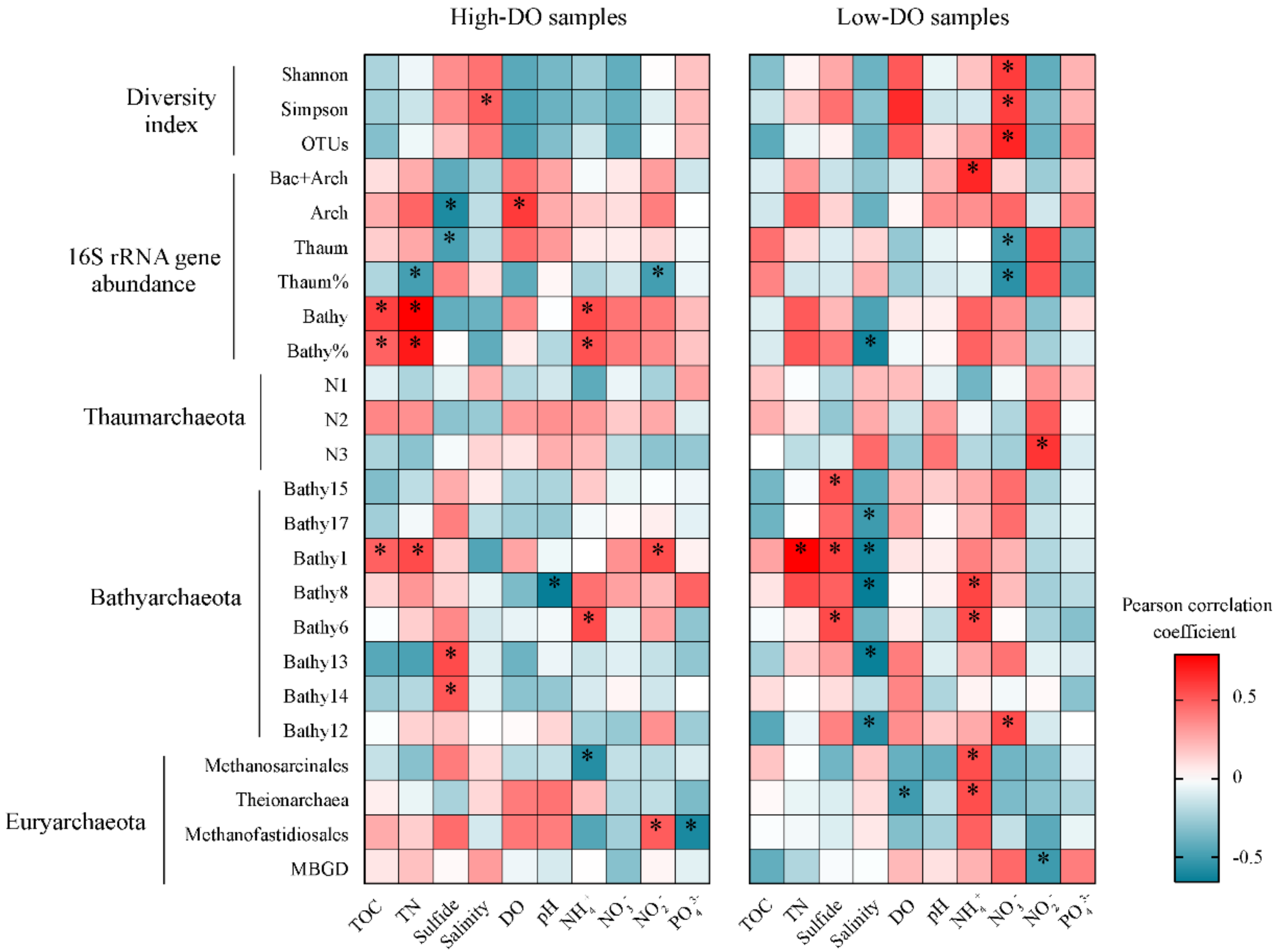

Figure S10 The heatmap of Pearson correlation coefficient between the environmental factors and the archaeal community properties, including the diversity index, the 16S rRNA gene abundance, and the major groups of *Thaumarchaeota*, *Bathyarchaeota*, and *Euryarchaeota*. The symbol * indicates that the correlation is significant ($p < 0.05$).

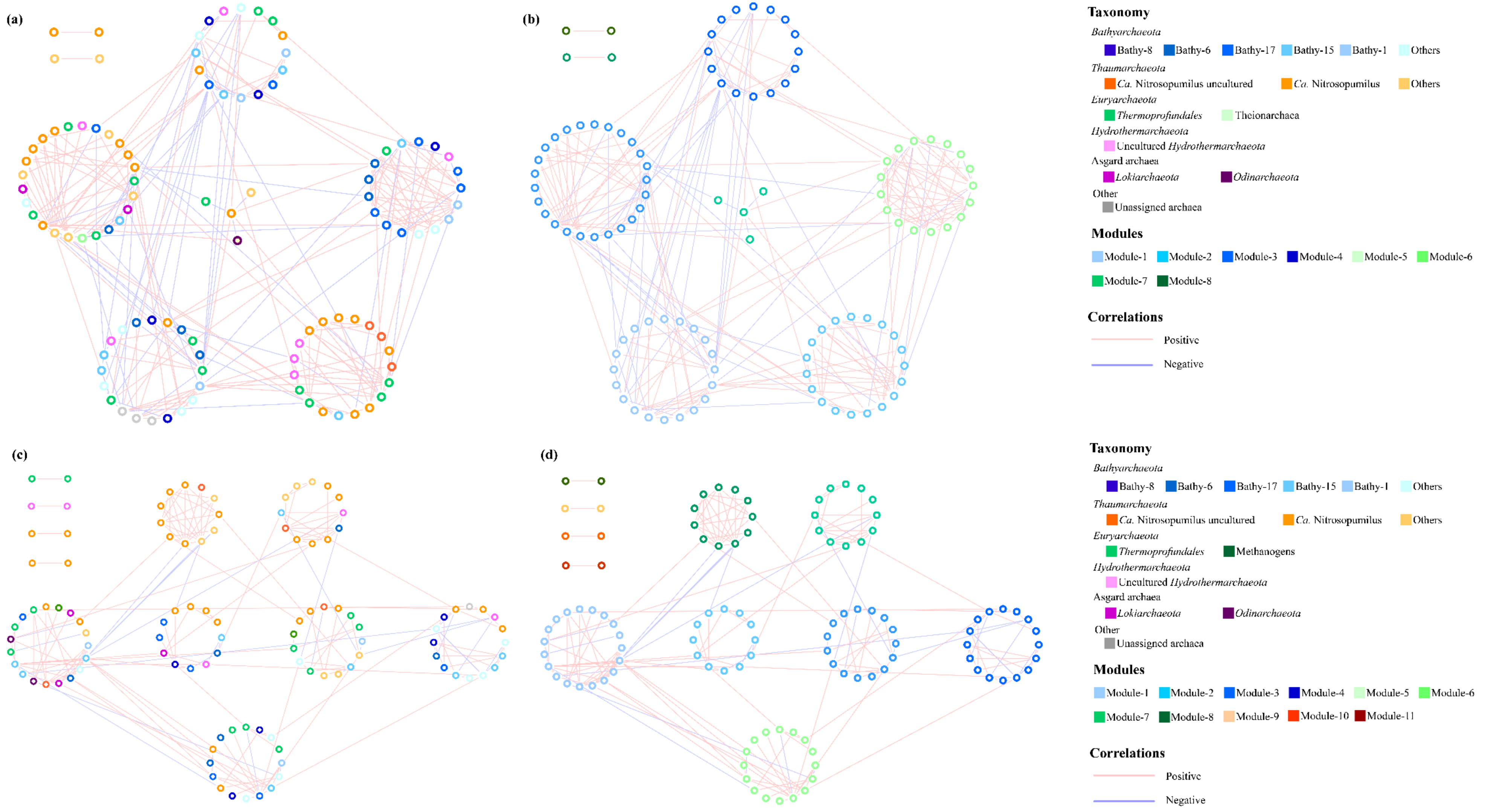

Figure S11 The network of co-occurrence patterns between major archaeal OTUs in the low- (a) and high-DO (c) samples. Predicted microbial modules were illustrated of the low- (b) and high-DO (d) samples. OTUs observed in ≥ 6 and ≥ 10 samples, and interactions with $p < 0.01$ were employed in network analysis for the low- and high-DO network, respectively. The low-DO network consisted of 105 nodes and 265 edges, with 8 modules. The high-DO network consisted of 109 nodes and 191 edges, with 11 modules.

References

Zhao, L., Wang, R., Zhang, C., Yin, D., Yang, S., & Huang, X. (2019). Geochemical controls on the distribution of mercury and methylmercury in sediments of the coastal East China Sea. *Science of the Total Environment*, *667*, 133-141.

Lin, X., Hou, L., Liu, M., Li, X., Zheng, Y., Yin, G., ... & Jiang, X. (2016). Nitrogen mineralization and immobilization in sediments of the East China Sea: Spatiotemporal variations and environmental implications. *Journal of Geophysical Research: Biogeosciences*, *121*(11), 2842-2855.

Liu M, Xiao T, Wu Y, Zhou F, Huang H, Bao S, et al. Temporal distribution of bacterial community structure in the Changjiang Estuary hypoxia area and the adjacent East China Sea.Environmental Research Letters 2012; 7: 025001.

Wang, M., & Gao, L. (2022). New Insights Into the Non-Conservative Behaviors of Nutrients Triggering Phytoplankton Blooms in the Changjiang (Yangtze) River Estuary. *Journal of Geophysical Research: Oceans*, *127*(2), e2021JC017688.
